# Implementation Status of Low-Dose Aspirin and Calcium Supplementation to Prevent Preeclampsia in Burkina Faso, Ethiopia, Kenya, Nigeria and Pakistan

**DOI:** 10.1007/s10995-026-04270-3

**Published:** 2026-06-03

**Authors:** Patricia S. Coffey, Kimberly Mansen, Nesibu Agonafir, Gbenga Ishola, Basilia Coefe Nitiema, Megan E. Parker, Soha Randhawa, Janet Shauri, Katharine D. Shelley

**Affiliations:** 1 PATH, Seattle, WA USA; 2 PATH, Addis Ababa, Ethiopia; 3 Independent Consultant, Abuja, Nigeria; 4 Independent Consultant, Ouagadougou, Burkina Faso; 5 Precision Health Consultants (PHC) Global, Karachi, Pakistan; 6 PATH, Nairobi, Kenya

**Keywords:** Preeclampsia, Low-dose aspirin, Calcium supplementation, Burkina Faso, Ethiopia, Kenya, Nigeria, Pakistan

## Abstract

**Purpose:**

Hypertensive disorders of pregnancy are a leading cause of maternal death. Global guidance recommends use of low-dose aspirin and calcium supplementation for prevention of preeclampsia. Published literature showcasing the implementation of these guidelines in low- and middle-income countries (LMICs) is sparse.

**Description:**

The objective of this practice note is to share our findings related to implementation of low-dose aspirin and calcium supplementation for the prevention of preeclampsia in Burkina Faso, Ethiopia, Kenya, Nigeria and Pakistan. The experiences from these five countries can illustrate important aspects about the nature and scope of scale of these two evidence-based interventions in LMICs.

**Assessment:**

Between May 2023 and March 2024, we conducted policy review and key informant interviews (*n* = 59) at global and country level. Implementation efforts for the two interventions show variations in the policy environment and clinical practices across countries. Progress toward scale of low-dose aspirin is hampered by variable clinical practices, low provider and community awareness, late antenatal care attendance, and under resourced health systems including weak supply chains. In the case of calcium supplementation, a complicated regimen and conditional global recommendations leaves countries without actionable plans aligning with optimal counseling models for improved adherence.

**Conclusion:**

Effective implementation of low-dose aspirin and calcium supplementation for preeclampsia prevention will require strengthening antenatal care and provision of services at the primary health care level and enabling the policy environment where needed. Increased coordination around a package of interventions aimed at reducing preeclampsia will also be critical going forward. Practice recommendations on policy, demand, clinical implementation, and monitoring are summarized.

**Supplementary Information:**

The online version contains supplementary material available at 10.1007/s10995-026-04270-3.

## Significance

What Is Already Known on This Subject?

What This Study Adds?

Use of low-dose aspirin and calcium supplementation are recommended by the World Health Organization and other normative bodies for prevention of preeclampsia. Documentation of these practices in low- and middle-income countries is sparse.

Application of the Stages of Achieving Effective Coverage and Equity framework to assess implementation status of a specific intervention can illuminate necessary next steps towards achieving equitable coverage. Near-term actions to advance low-dose aspirin and calcium supplementation towards scale are delineated for five countries (Burkina Faso, Ethiopia, Kenya, Nigeria and Pakistan).

**Supplementary Information:**

The online version contains supplementary material available at 10.1007/s10995-026-04270-3.

## Introduction

The rate of maternal mortality across the world remains unacceptably high. According to the World Health Organization (WHO), in 2020, an estimated 287 000 women globally died from a maternal cause, equivalent to almost 800 maternal deaths every day or one every two minutes. Almost all of these maternal deaths occurred in low and lower middle-income countries (LMICs) yet almost 95% of these deaths are thought to be preventable (World Health Organization 2023a, 2023b, 2024). Hypertensive disorders of pregnancy (i.e., hypertension, preeclampsia and eclampsia) are a leading cause of maternal death and account for about 14% of maternal mortality globally (Say et al., 2014). Data from the 2023 Global Burden of Disease report estimate that the global prevalence of hypertensive disorders of pregnancy is 72.6 per 100,000 women of childbearing age with the highest mean prevalence of 183.0 per 100,000 women of childbearing age in the Sub-Saharan Africa region (Institute for Health Metrics and Evaluation (IHME), 2025).

Preeclampsia affects 2–5% of pregnant people usually from around 20 weeks gestation and is estimated to account for 76,000 maternal deaths and 500,000 neonatal deaths globally every year (FIGO, 2019). High-quality evidence from the ASPRE trial showed the incidence of preterm preeclampsia was reduced by 62% when low-dose aspirin was administered to pregnant women beginning in their first trimester *(*Rolnik et al., 2017). Preventive therapy with calcium has been shown to reduce the risk of preeclampsia by almost half, especially in populations with low dietary intake *(*Hofmeyr et al., 2018). Based on a review of recent evidence, the WHO and other normative bodies have issued guidance on preventing preeclampsia through use of low-dose aspirin (75–150 mg) for women at moderate to high risk of preeclampsia, and calcium supplementation (1.5–2.0 g of elemental calcium daily spread over three doses per day) during pregnancy for populations with low dietary intake (Sinkey et al., 2020; World Health Organization 2011, 2018, 2021). In a 2022 survey among 31 LMICs, a majority of countries reported that guidelines around prevention of preeclampsia had been updated with recent recommendations about use of low-dose aspirin (74%) and calcium supplementation (65%) (Noriega et al., 2022). Also, around 70% of the 31 LMICs indicated that their pre-service and in-service curricula were updated to align with these global recommendations. Yet, published literature showcasing the implementation of these guidelines in LMIC is sparse.

The purpose of this investigation was to track the journey to scale for key maternal, newborn, child health and nutrition (MNCHN) evidenced-based best practices. The objective of this practice note is to share our findings related to implementation of low-dose aspirin and calcium supplementation for the prevention of preeclampsia in Burkina Faso, Ethiopia, Kenya, Nigeria and Pakistan. The five countries, which were selected based on funder interest, represent varying degrees of health system decentralization from formally devolved structures with substantial subnational authority (Ethiopia, Kenya, Nigeria, Pakistan) to a more centralized system (Burkina Faso), and their experiences can illustrate important aspects about the nature and scope of scale of these two evidence-based interventions in LMICs. As awareness is being raised around best practices for addressing preeclampsia in countries, more clarity around what is needed to advance scale of low-dose aspirin and calcium supplementation, two high-impact interventions to prevent preeclampsia, could be beneficial to improve clinical care.

## Methods

This exploratory, descriptive, mixed methods assessment aggregated data from policies, guidelines, surveys, national health management information systems, and key informant interviews to understand the status of scale of 22 MNCHN evidenced-based best practices; this paper presents results related to only two of these practices: low-dose aspirin and calcium supplementation for the prevention on preeclampsia. We grounded our assessment in a six-stage theoretical framework that shows how an intervention (or practice) moves from introduction towards equitable, effective coverage at country level. The Stages of Achieving Effective Coverage and Equity framework includes key milestones typically achieved on the pathway to scale beginning from the enabling policy environment to health system integration and readiness to implementation and service delivery to availability and, ultimately, coverage (including effective and equitable coverage) (Coffey et al., 2025).

To do this, we performed rapid literature review and data mapping​, collated secondary data for milestones aligned with the scale up framework, conducted global-level and country-level key informant interviews to contextualize scale up progress and garner recommendations, triangulated and synthesized these data, and developed dashboard visualizations. The full framework and underlying dataset can be accessed through an interactive, web-based Tableau dashboard for data visualisation and exploration (PATH, 2024). Document review included key global and country-specific policy documents, guidelines, and peer-reviewed and grey literature. Data for indicator mapping came from review of global and national surveys (de-identified data) to identify data sources and available data for key indicators and milestones aligned to the scale up framework.

Key informant interview participants were identified through purposive sampling of global and country- based experts. These included global and national-level technical, policy, and programmatic leaders with expertise related to the intervention of interest who were over the age of 18 years. Key stakeholders in focus countries include MOH officials, technical partners, health professional associations, academic researchers, health care providers, and civil society organizations and advocates. Interview guides primarily focused on understanding barriers and enablers to scale up, and filling gaps in indicators related to the scale up framework that could not be sourced elsewhere. Interviews were conducted virtually for global-level experts and both in-person and virtually for national-level experts; the project team, with expertise in maternal, newborn, child health and nutrition, conducted all interviews. We obtained verbal consent from all key informant interview respondents and typically completed interviews within one hour. Interviewers transcribed detailed notes during each interview, which were subsequently expanded, cleaned, and entered in a structured Smartsheet (Bellevue, USA) data processing template for data management and analysis. We used a thematic analysis approach based on the stages of the framework to analyze the qualitative data derived from multiple sources. The results of near-term actions were created from the qualitative data by the project team and in some cases co-created with in-country partners. These draft actions were reviewed and validated at national level dissemination meetings by key informants in each country.

This research was conducted in accord with prevailing ethical principles. The assessment (Sponsor Protocol Number RES-00534) underwent ethics review and was granted an exemption by WCG IRB (USA) in April 2023 and the protocol was also approved (Ref. Number ERC/S20/P-026) by the Applied Economics Research Centre (AERC) Committee on Ethics IRB in Pakistan in May 2023 and the Maseno University Ethics Review Committee in Kenya in August 2023 (included within an existing umbrella protocol Ref. Number 1628333 [IRBNet]). For the other three countries, local IRB review was not required and instead we obtained a letter of approval from the Ministry of Health to conduct all data collection activities.

## Assessment Results

Between May 2023 and March 2024, we conducted key informant interviews with a total of 59 stakeholders including at the global level (*n* = 12) and from Burkina Faso (*n* = 12), Ethiopia (*n* = 8), Kenya (*n* = 8), Nigeria (*n* = 6) and Pakistan (*n* = 13). The status of current implementation efforts for low-dose aspirin (Table 1) and calcium supplementation (Table 2) shows variations in the policy environment and clinical practices across countries. Progress toward scale of low-dose aspirin is hampered by variable clinical practices, low provider and community awareness, late antenatal care attendance, and under resourced health systems.​ In the case of calcium supplementation, a complicated regimen and conditional global recommendations leaves countries without actionable plans aligning with optimal counseling models for improved adherence (see Supplemental File 1 for additional country-specific details on methods, data sources, and results summaries).

**Table 1:** Implementation status of low-dose aspirin for prevention of preeclampsia, by country

Low-dose Aspirin	nEML	National policy	Target population	Training	Screening modality	Treatment regimen (dose)	Data availability	Product availability
Burkina Faso	Yes	Yes ^1^	Pregnant women at high risk of PE	Not included	Maternal history, blood pressure	Daily tablet (125 mg) from 14–36 weeks gestation	Women with PE/E reported in HMIS; no relevant indicators on product use	Only 500 mg aspirin tablet available in public sector supply chain so patients are advised to split tablet into four pieces
Ethiopia	Yes*	Yes ^2^	Pregnant women at high risk of developing PE	Included in pre- and in-service training curricula	Maternal history, blood pressure, and risk assessment	Clinical guidelines being revised with dosing updated (from 81 mg to 75 mg)	Women with PE/E reported in HMIS; no relevant indicators on product use	Two locally manufactured products available on market:
								• Aspi-SSP 81: 81 mg tablets,10 tablet strip (Sansheng Pharmaceutical PLC, Oromia, Ethiopia) (Ethiopian Food and Drug Administration 2023)
								• E-SPIRINS: 81 mg tablets, 10 tablet blister (Ethiopian Pharmaceuticals Manufacturing Sh. Co (EPHARM), Addis Ababa, Ethiopia) (Ethiopian Pharmaceuticals Manufacturing, 2016)
Kenya	Yes	Yes ^3^	Pregnant women at high risk of developing PE	Included in pre- and in-service training curricula	Maternal history, presence of chronic disease (diabetes, kidney disease, hypertension)	Nightly tablet (150 mg) from 11–14 + 6 until 36 weeks gestation or until delivery or when preeclampsia is diagnosed.	Women with PE/E reported in HMIS; no relevant indicators on product use	75 mg aspirin available in public sector supply chain
Nigeria	Yes*	Yes ^4^	Pregnant women at high risk of developing PE	Included in pre- and in-service training curricula	Clinical guideline being developed	Clinical guideline being developed (75 mg)	Women with PE/E reported in HMIS; no relevant indicators on product use	One locally manufactured product available on market: 300 mg generic aspirin tablet, (Juhel Pharmaceuticals, Enugu, Nigeria) (JUHEL, 2022)
Pakistan	Yes*	Yes ^5^	Pregnant women at high risk of PE	Included in pre- and in-service training curricula	Maternal history, blood pressure, proteinuria test	Nightly tablet (75 mg) starting before 20 (and if possible as early 12) until 36 weeks gestation	Women with PE/E reported in HMIS; no relevant indicators on product use	Two locally manufactured drugs on market:
								• Loprin: 75 mg enteric coated tablets, 30 per pack (Highnoon, Lahore, Pakistan) (Highnoon, 2022)
								• A pack of 30 tablets of 75 mg Ascard; 75 mg enteric coated tablets, 30 per pack (Atco, Karachi, Pakistan) (ATCO Lab, 2022)

Abbreviations: *HMIS* health management information system; *mg* milligram; *nEML* national essential medicines list; *PE* preeclampsia; *PE/E* preeclampsia/eclampsia

*no pre-eclampsia indication

^1^ Burkina Faso policy: Protocoles de Santé de la reproduction : Santé de la femme et du nouveau-né de moins de 7 jours (2018) [Reproductive Health Protocols: Health of Women and Newborns Under Seven (7) Days of Age (2018)]

^2^ Ethiopia policy: National Antenatal Care Guidelines (2022)

^3^ Kenya policy: National Guidelines on Quality Obstetrics and Perinatal Care (2022)

^4^ Nigeria policy: National Guidelines for Management and Treatment of Eclampsia and Pre-Eclampsia (2024), pending approval by the Honorable Minister for Health and Social Welfare

^5^ Pakistan policy: The National Comprehensive SRHR Guidelines 2022 (Baqai et al., 2022)

**Table 2:** Implementation status of calcium supplementation for prevention of preeclampsia, by country

Calcium	nEML	National policy	Target population	Training	Treatment regimen (dose)	Data availability	Product availability
Burkina Faso	Yes*	No ^6^	n/a	Not included	n/a	HMIS: Women with PE/E reported in HMIS; no relevant indicators on product use FAO^11^: No national survey available, but subnational data from 2010 including women of reproductive age in rural areas, indicating average dietary intake of calcium was low at 497 mg/day.	Yes, available in various formulations, such as IDEOS Calcium Carbonate 500 mg (30 tablets per package).
Ethiopia	Yes*	Yes ^7^	All pregnant women	Included in pre- and in-service training curricula	Daily calcium supplementation during pregnancy: starting from 14 weeks of gestation (1.5–2.0 g oral elemental calcium)	HMIS: Women with PE/E reported in HMIS; no relevant indicators on product use FAO^11^: No national survey available; one subnational survey (2013) including mothers and children in rural areas showed calcium intake 363 mg/day; second survey (2013) in pregnant and non-pregnant women in rural areas indicated low calcium intake averaging 268 mg/day	Yes, available in various formulations such as CalciD-Denk, 1,000 mg Calcium Carbonate + 22 micro grams vitamin D3 (20 tablets per package). In-country market authorization identified for five products although none are locally manufactured (eRIS, EFDA 2023).
Kenya	Yes*	Yes ^8^	Pregnant women with low dietary intake of calcium and/or any pregnant woman with high risk for PE** regardless of calcium dietary intake	Included in pre- and in-service training curricula	For women with low dietary calcium intake recommend starting first trimester, or prior to 16 weeks. (at least 1 g/day orally)	HMIS: Women with PE/E reported in HMIS; no relevant indicators on product use FAO^11^: No national datasetavailable. Six subnational datasets from 2012 (rural and urban, children and mothers, average 364 mg/day, 2014 (sub analysis of rural adults averaging low intake at 441 mg/day). 2014 (sub analysis of rural adults averaging low intake at 541 mg/day), 2015 (sub analysis of rural adults averaging 411 mg/day), 2016 (sub analysis of rural adults averaging low intake at 155 mg/day), 2018 (sub analysis of rural adults averaging sufficient intake 1008 mg/day)	Yes, available in private sector in various formulations such as Lamberts Chewable Calcium 400 mg + Vitamin D (60 tablets per package).
Nigeria	Yes*	Yes ^9^	Populations with low dietary calcium intake, pregnant women should receive calcium supplementation	Included in pre- and in-service training curricula	Timing not prescribed in ANC guidelines (1.5–2.0 g oral elemental calcium)	HMIS: Women with PE/E reported in HMIS; no relevant indicators on product use FAO^11^: No national data set available. Subnational dataset (2011) for calcium intake in women of childbearing age in rural and urban areas shows low average calcium intake of 374 mg/daily)	Yes, widely available in a variety of formulations such as Ca-C1000 Calcium 260 mg.
Pakistan	Yes*	No ^10^	n/a	Included in pre- and in-service training curricula	n/a	HMIS: HMIS: Women with PE/E reported in HMIS; no relevant indicators on product use FAO^11^: No national dataset available. One subnational dataset from 2014 of first trimester rural pregnant women averaging low intake of 543 mg/day.	Yes, widely available in a variety of formulations, such as QalSium-D Calcium 1,250 mg Calcium Carbonate + Vitamin D3.

Abbreviations: *ANC* antenatal care; *FAO* Food and Agricultural Organization; *g* gram; *HMIS* health management information system; *mg* milligram; *n/a* not applicable; *nEML* national essential medicines list; *PE* preeclampsia; *PE/E* preeclampsia/eclampsia

*no preeclampsia indication

** See Table 1 for screening methods used to determine high risk

^6^ Burkina policy: None

^7^ Ethiopia policy: Reproductive Health Strategic Plan 2021–2025, National Antenatal Care Guideline (2022), Obstetric Management Protocol for Hospitals (2021)

^8^ Kenya policy: National Guidelines on Quality Obstetrics and Perinatal Care (2022)

^9^ Nigeria policy: 2018 National Guideline on ANC orientation package

^10^ Pakistan policy: None

^11^*FAO* national/subnational food consumption data availability (FAO/WHO GIFT n.d.)

It is imperative that these two lifesaving best practices translate effectively into clinical practice. The next immediate actions to advance the scale of low-dose aspirin and calcium supplementation (Table 3) to prevent preeclampsia in each of the five countries relate primarily to strengthening the enabling policy environment with longer term effort directed towards system integration and readiness, and implementation and service delivery.

**Table 3:** Near term actions to advance the scale up of low dose aspirin and calcium supplementation

Country	Stage of scale up framework			
	National policy adoption	System integration and readiness	Implementation and service delivery	Availability and Coverage
Burkina Faso	Low dose aspirin			
	• Include low dose aspirin on the national EML for the indication of preeclampsia prevention • Work with donors and implementers to develop training modules and job aids • Encourage national quantification for low-dose aspirin for the prevention of preeclampsia/eclampsia	• Prioritize inclusion of low dose aspirin in pre-service and in-service training modules	• Continue to encourage attendance at prenatal consultations, particularly in the first trimester, and strengthen counseling around hypertensive disease of pregnancy.	• None identified
	Calcium supplementation			
	• Obtain recent data on the calcium status of pregnant women in the country and share the results widely • Support a national discussion on the integration of calcium supplementation into policies and protocols​ • Based on this decision, update protocols to emphasize the use of calcium for preeclampsia prevention • Include on national EML with the indication of prevention of preeclampsia	• None identified	• None identified	• None identified
Ethiopia	Low dose aspirin			
	• Ensure the national EML lists low dose aspirin (75–81 mg) for prophylaxis for pregnant women at moderate to high risk of preeclampsia​ • Ensure quantification and allocation of budget for low dose aspirin for women at risk of preeclampsia	• Evidence-based advocacy targeting national and subnational stakeholders to support financing of scale up of low dose aspirin • Develop and avail job aid to service providers​	• Build capacity of ANC service providers through training and mentorship and distributing ANC guidelines, especially at PHC level • Strengthen demand creation for ANC to increase early care seeking, possibly by engaging religious leaders or traditional healers in increasing preeclampsia awareness (Robbins et al. 2021)	• Incorporate low dose aspirin indicators into HMIS, LMIS, and other relevant surveys to assess adherence and monitor service delivery and measure coverage
	Calcium supplementation			
	• Update national EML to list calcium supplementation (1.5–2.0 g of elemental calcium daily spread over three doses per day) for the prevention of preeclampsia. • Analyze health facility reports (and other data sources) of increasing maternal deaths from hypertension and use evidence to inform decision-making around calcium supplemenation scale up • Ensure forecasting, costing, and budget allocation for calcium	• Evidence-based advocacy targeting national and subnational stakeholders to support financing of scale up of calcium supplementation	• Train service providers and ensure efficient use through targeting high-risk groups	• Incorporate calcium indicators into HMIS, LMIS, and other relevant surveys to assess adherence and monitor service delivery and measure coverage
Kenya	Low dose aspirin			
	• Update national EML to incorporate use of low dose aspirin for prevention of preeclampsia and lower the level of use to level 2 facilities​.	• None identified	• Disseminate guidelines to sub-county and facility level.​ • Initiate HCW training and mentorship for nurses and clinical officers.	• Expand access to point of care ultrasound machines in lower-level facilities to support gestational age dating
	Calcium supplementation			
	• Identify a dedicated team from Division of Reproductive and Maternal Health and Division of Nutrition to examine opportunities for scale, including engaging in policy dialogue to build case for introduction and scale of calcium supplementation • Use advocacy to elevate need to address eclampsia as a major cause of maternal death among government stakeholders • Update the Emergency Obstetrics and Newborn Care Guidelines (EmONC) and pre-service and in service curricula (2022) to incorporate use of calcium supplementation in preventing preeclampsia during pregnancy to align with WHO 2018 recommendations (of 1.5–2 g daily with a caution of iron interaction)	• None identified	• Disseminate national guidelines to the counties and facility level. • Initiate HCW training, mentorship, and develop job aids to ensure effective utilization	• Create more cost-effective options in packaging with appropriate design
Nigeria	Low dose aspirin			
	• Update national EML to specify preeclampsia indication​ for low dose aspirin	• Update pre-service and in-service training manual to incorporate the use of low dose aspirin to prevent preeclampsia during pregnancy ​	• Work with donors and implementing partners to implement training manual and job aids​	• None identified
	Calcium supplementation			
	• Update national EML to specify preeclampsia/eclampsia indication for calcium supplementation	• Update pre-service and in-service training manual to incorporate the use of calcium supplementation to prevent preeclampsia during pregnancy	• Work with donors and implementing partners to implement training manual and job aids • Raise provider and community awareness about use of calcium to prevent preeclampsia as distinct from use for pregnant women with calcium deficiency	• None identified
Pakistan	Low dose aspirin			
	• Include low dose aspirin (75, 150 mg) in national EML and provincial EMLs with specific indication of preventing preeclampsia • Revise low dose aspirin use/indications on product bottle to include prevention of risk of preeclampsia (to reduce off-label use)​ • Develop a costed implementation plan for low dose aspirin use for preeclampsia prevention • ​Develop a demand calculation mechanism for low dose aspirin (specifically for preeclampsia) and related equipment and resources	• Develop job aids and preeclampsia screening checklists to support proper use and accurate prescription of low dose aspirin until prophylactic use is approved	• None identified	• Ensure supply of necessary supportive services (doppler, blood pressure cuff); they must be regulated to avoid wastage of resources • Streamline availability of low dose aspirin (75–81/150 mg) across private and public health facilities ​ • At the Federal level, improve supply chain management systems to address bottlenecks leading to inflated drug prices
	Calcium supplementation			
	• Include elemental calcium in national EML and provincial EMLs with indication of preeclampsia prevention • Revise national policy to include clear language on preeclampsia prevention to include elemental calcium intake of 1.5–2.0 g divided into three doses daily • Invest in national clinical trials to document the effectiveness of elemental calcium in preventing preeclampsia among women living in areas with low calcium intake (prevention vs. treatment)	• Update training curricula of lady health workers and community midwives to include calcium supplementation for prevention of preeclampsia. Currently, the curriculum only discusses its use as dietary supplement and for bone strengthening.	• Engage in community awareness sessions on the importance of elemental calcium for the prevention of preeclampsia in areas with reported low-calcium intake • Enhance community confidence in public health centers and providers to ensure adherence to calcium supplement intake	• Subsidize provision of calcium supplements to rural areas due to lower calcium intake and income levels • Make multiple micronutrient tablets with calcium supplementation more available

The original framework has 6-stages (Coffey et al., 2025)—as this table is focused on country-specific near term actions, we have dropped the first stage of the framework (global guidelines), and merged availability and coverage for space reasons

Abbreviations: *ANC* antenatal care; *EML* essential medicines list; *EmONC* Emergency Obstetrics and Newborn Care; *g* gram; *HCW* health care worker; *HMIS* health management information system; *LMIS* logistics management information system; *PHC* primary health care; *WHO* World Health Organization

The immediate next steps at country level should also consider some enablers to scale that have to do with refinements in implementation options. For example, a multi-country study of systematic preeclampsia risk screening in Africa, which will compare the efficacy of 75 vs.150 mg low-dose aspirin for preeclampsia prevention in Ghana, Kenya and South Africa is being launched (Ekman 2024). In terms of screening options to define the women at risk of preeclampsia and in need of low dose aspirin, the Fetal Medicine Foundation offers a free, on-line risk calculator that provides a clear numerical estimation of individual risk (The Fetal Medicine Foundation n.d.). This can be helpful because women may possibly be more likely to take low-dose aspirin if given a quantified risk estimate. Also, an affordable angiogenic biomarker point of care placental growth factor PlGF lateral flow screening diagnostic being developed on the portable RONIA™ Platform that uses a finger prick blood sample could offer an opportunity to integrate an affordable angiogenic biomarker test for preeclampsia into antenatal care (Revvity, 2024).

Recent data from India and Tanzania have shown the noninferiority of a 500-mg daily dose to a 1500-mg daily dose of calcium supplementation when used for preeclampsia prevention (Dwarkanath et al., 2024). This opens the way for a more feasible dosing regimen and bodes well for implementation (Hodgins & Mathai, 2024). Guidance from normative bodies such as WHO could consider using these data to update existing recommendations with the aim of tailoring a feasible programmatic intervention that aligns with limited local dietary consumption information. Recently, an updated Cochrane review found that calcium supplementation in pregnant women has no effect on the prevention of pre-eclampsia (Cluver et al., 2025). These results may impact future global guidance but given the benefits of calcium supplementation in pregnancy beyond the question of pre-eclampsia, the findings presented here can still be relevant to support nutritional supplementation programs in pregnant women.​

## Conclusions for Practice

Generally, effective implementation of low-dose aspirin and calcium supplementation for preeclampsia prevention will require strengthening antenatal care and provision of services at the primary health care level and enabling the policy environment where needed. Increased coordination around a package of interventions aimed at reducing preeclampsia will also be critical going forward.

Specifically, efforts could focus on:

- Creating a robust policy framework where needed taking note that updating the national essential medicines list to include an indication for use of the two interventions for preeclampsia prevention may be required.
- Strengthening demand creation for antenatal care to increase early care seeking, possibly by enhancing communication/counseling around hypertensive diseases of pregnancy at the community level. In Ethiopia, a promising approach was engaging religious/traditional healers in shifting community awareness of preeclampsia/eclampsia (Robbins et al. 2021).
- Developing clearer clinical flowcharts within antenatal care guidelines on the relationship between these two interventions, as well as integrated messaging for providers, including community health workers, to counsel women identified at moderate to high risk of preeclampsia on the importance of both calcium supplementation and low dose aspirin.
- Integrating point of care ultrasound devices (possibly including AI-enabled devices) and other new and underutilized screening technologies into antenatal care to allow for accurate gestational age estimation. Experiences in Kenya suggest feasibility of use of obstetrics point-of-care ultrasound for high-risk conditions in rural public healthcare facilities (Vinayak & Brownie, 2018; Wachira et al., 2023).
- Prioritizing delivery of calcium as part of existing nutritional supplementation programs (i.e., iron-folic acid and/or multiple micronutrient) being distributed through antenatal care, and monitored by community health workers, in order to improve counseling and adherence to daily dosing. In areas of known low dietary calcium, community health workers have also been proposed as a potential distributor where early antental care access is uncommon (World Health Organization 2018).
- Monitoring uptake and coverage of the two interventions at a population-based level, possibly by including a subset of indicators in existing population-based or facility-based surveys. Options might include integration of a module around the preeclampsia continuum of care into the Demographic and Health surveys, WHO Service Availability and Readiness Surveys (SARA), or Global Financing Facility FASTR pulse surveys to enable rapid cycle analytics and data use.

Various efforts are currently being undertaken to address postpartum haemorrhage, which accounts for about one-quarter of all maternal death globally. In particular is the creation of the WHO Roadmap to Combat Postpartum Haemorrhage between 2023 and 2030, which lays out an action-oriented global agenda across the areas of norms and standards, research, implementation and advocacy (World Health Organization 2023b). This type of coordinated focus that includes various stakeholders from global to community that can act as a catalyst to reducing the burden of maternal mortality related to postpartum hemorrhage may serve as a model for hypertensive disorders of pregnancy as well. In 2026, the first global pre-eclampsia summit hosted by WHO and the Human Reproduction Programme (HRP) will serve as a catalytic platform for aligning and coordinating key actions related to hypertensive disorders of pregnancy through 2030 and beyond (World Health Organization, 2026).

## Supplementary Information

Below is the link to the electronic supplementary material.

Supplementary Material 1

## Data Availability

The data dashboards are publicly available online: Tracking the journey to scale for lifesaving maternal, newborn, and child health and nutrition interventions | Primary health care | PATH.
