# Supplementary material for: Implementation Status of Low-Dose Aspirin and Calcium Supplementation to Prevent Preeclampsia in Burkina Faso, Ethiopia, Kenya, Nigeria and Pakistan
Source: Matern Child Health J. 2026 Jun 3;30(6):731–41. doi: 10.1007/s10995-026-04270-3 (PMC13275748; doi:10.1007/s10995-026-04270-3)
Supplement: Supplementary file 1 — Supplementary Material 1 [file 10995_2026_4270_MOESM1_ESM.pdf]

Implementation status of low-dose aspirin and calcium supplementation to prevent preeclampsia in Burkina Faso, Ethiopia, Kenya, Nigeria and Pakistan

Supplemental File 1

Details of country-specific methods, data sources, and findings

Asset Tracker Framework

ramework: we adapted existing scale-up frameworks to propose a six
age framework that ***moves closer to equitable, effective coverage***

Global guidelines & market availability	National policy adoption	System integration & readiness	Implementation & service delivery	Availability	Coverag
O recommendation a national or global lition of partners ablished.	Numerous steps from national policy adoption to regulatory body approval to costed implementation plan and budget line adopted.	In-service and on the job training curricula/manual updates, indicators for measurement, and procurement.	Numerous steps at sub-national and community level including guidelines dissemination, training conducted, demand generation activities.	Availability of assets and job aids where people receive care.	Populatio coverage and equit

- spite marked improvements in MNCH outcomes globally, progress has been slow and uneven.
- nough innovations exist around many essential, evidence-based MNCHN interventions, significant g nain in their universal coverage.
- **is analysis aggregates multiple data sources to examine the pathway to scale for priority erventions or "Assets", including lifesaving commodities, tools, and approaches.**

PA

Burkina Faso

Méthodes et sources de données

éthodologie pour les entretiens vec les informateurs clés et analyse

Un guide d'entretien structuré, organisé en fonction des étapes théoriques du passage à l'échelle a été utilisé

Des entretiens en présentiel et virtuels ont été conduits entre Juin et Juillet 2023 avec des représentants d'organisations ciblées

Les participants ont donné leur consentement verbal

Des notes manuscrites et électroniques ont été prises, puis transcrites électroniquement et entrées dans un document Smartsheet, avant d'être exportées dans un canevas pour favoriser les analyses croisées par thème important

Certaines organisations ont partagé des rapports programmatiques additionnels, de la littérature scientifique, et autres documents importants après les entretiens

Liste des organisations enque

- Direction de la Santé de la Famille (MOH Famil
- Institut de Recherche en Sciences de la Santé (Research on Health Sciences) (IRSS)
- Association de sages femmes
- IntraHealth
- Global Financing Facility (GFF)
- Centrale d'Achat des Médicaments Essentiels G (Drugs Procurement Agency) (CAMEG)
- Direction Générale de l'Accès aux Produits de Santé (Direction for access to health products) (
- Pathfinder
- JHPIEGO
- Société Burkinabe de Pédiatrie/Néonatologie
- USAID
- UNFPA
- Société de Gynécologie et d'Obstétrique du Bur
- UNICEF

ésumé des sources de onnées

Nombre total de documents revus : 29

Nombre total d’entretiens avec informateurs clés conduits (n=16), et par type d’informateurs :

- ❖ Agences de plaidoyer = 8
- ❖ Agences de prise de décision = 4
- ❖ Agences de mises en œuvre = 4

mé des sources de données

e total de documents revus:

ources	Nombre total de documents revus pour toutes les 8 interventions
	**10** (7 (pour les coalitions mondiales)
ns	3 (TXA, CPAP, CAMMS)
(s) de Politique National	**4** (SONU+ 1 PCIME+ Santé de la femme et du nouveau né + plan stratégique intégré SRMNIA)
	**3** documents (y compris les aides mémoires pour les CPN, counseling/prise en charge des complication, guide PCIM SONU)
MEG	I listing CAMEG
	1 Liste Nationale des Médicaments Essentiels Génériques et des consommables médicaux
ckout reports	1 SARA
nationales	**1** EDS
de formation	**5** documents (PCIME, PCIME communautaire, SONUC prééclampsie et prématurés)

Résultats

ina Faso: contexte

des soins aux mères et aux x nés (DHS: 2021)	Valeur de l'indicateur
onsultation prénatale avant 4 mois	53%
n de la grossesse en mois à la première visite re	3.9
atales pour la première grossesse	2.1%
atales pour la grossesse : 4+	72.1%
atales pour la grossesse : 8+	0.9%
ent assisté par du personnel qualifié	95.8%

Disponibilité des médicaments (SARA 2018)	Valeur d l'indicat
Aspirine (dosage non précisé) disponible	31%
Beta- or Dexaméthasone disponible	17%
Nifédipine disponible	46%
Azithromycine capsule/comprimés ou sirop disponible	4%
Zinc en stock	14%
ORS en stock	18%

'approvisionnement

e de collaboration entre les programmes travaillant à la mise en œuvre des interventions et la CAMEG à l'origine de rupture :
mes introduisent souvent les produits à travers une dotation initiale sans passer par la CAMEG, rendant le passage à l'échelle ultérieur avec la CAM
dispose d'un logiciel de gestion des stocks qui n'a pas d'interface ou de relation avec le Ministère de la Santé. Ils ne sont donc souvent pas informés d
oduits utilisés ou recommandés dans les formations sanitaires.
pourrait être de lier la CAMEG avec l'application NetSIGL2 (Softworks, Dhaka, Bangladesh), qui est une application sous la plateforme DHIS2

situations de ruptures pourraient être causées par des problèmes au niveau des formations sanitaires DMEGs :
- Les ruptures pourraient être liées aux faibles capacités de stockage des formations les empêchant de commander de grandes quantités
- Elles pourraient aussi être dues à l'insuffisance des ressources financières pour les commandes, liée aux difficultés de remb oursement des dépenses dans le cadre de la gratuité
- Si un produit n'est pas rentable, ne se vend pas assez, la formation sanitaire peut arrêter de le commander et cela peut être à l'origine de ruptures

SUPPLEMENTATION EN CALCIUM (SuppCa)

EFINITION DE L'INTERVENTION: Dans les régions où la consommation alimentaire de calcium est faible, les mmes enceintes à la première consultation prénatale de préférence (CPN1) recevront une prise quotidienne de 5-2.0 g éléments de calcium réparti en trois prises pour la prévention de la prééclampsie.

Facteurs favorisant le passage à l'échelle

Les femmes enceintes peuvent avoir facilement accès aux comprimés de calcium

Calcium (500 mg comprimés) est inscrit sur la liste nationale des médicaments essentiels (LME) (dans la rubrique Vitamines et sels minéraux), mais sans indication précise spécifiée

Les gynécologues-obstétriciens connaissent l'intervention mais, ce n'est pas une pratique courante de prescrire une supplémentation de calcium pour la prévention de la PE/E

Le Calcium très disponible dans le secteur pharmaceutique privé

En raison de l'essai clinique prochain sur le calcium, l'aspirine faiblement dose et les suppléments de multiple micronutriment CAMMS), l'intérêt des acteurs pour le calcium est croissant

"Une rencontre
gynécologues av
chercheurs de l'
pour échanger
l'importance d
supplémentatio
calcium dan
la prévention
prééclampsie a ét
(Agence de plaid
Burkina Fas

BARRIERES DU PASSAGE A L'ECHELLE

sence de recommandation nationale en raison des conditionnalités des commandations de l'OMS réduisant l'utilisation aux femmes enceintes ayant une mentation pauvre en calcium

Pas de politique nationale pour utilisation de la supplémentation en calcium pour la prévention PE/E

Les acteurs du ministère de la Santé ont noté l'insuffisance de données récentes sur la consommation de calcium par les femmes enceintes rendant difficile l'adoption d'une politique nationale sur la base de la recommandation actuelle de l'OMS

Des données de 2006 (Becquey et al: 2010) ont montré une consommation moyenne de calcium de 358 mg/jour pour les adultes Burkinabè pour un minimum d'apport journalier recommandé de 1000 mg pour les femmes âgées de 50 ans et moins

La consommation journalière de calcium pour les femmes enceintes Burkinabè n'est pas disponible, même si les données existantes tendent à montrer que les femmes au niveau de la population auraient tendance à ne pas consommer des quantités suffisantes (Becquey and Martin-Prevel:2010; Martin-Prevel et al:2010)

Le calcium comprimé n'est pas disponible à la CAMEG pour la distribution dans le secteur public quelle que soit l'indication

Seuls 25 % des établissements proposaient des conseils prénatals sur la supplémentation en calcium; moins de 2 % des établissements disposent de comprimés de calcium (HHFA 2021)

"N'est pas inscrit d
politiques, normes et
et il n'y a pas d'inten
faire actuellem

Agence de prise de
Burkina Fas

ogrès vers les étapes essentielles du passage à l'échelle

s étapes pour l'adoption nationale de la politique et la préparation du système ne sont pas remplies.

e pas de données de disponibilité ou de couverture pour suivre l'utilisation de la supplémentation en calcium par les femmes enceintes

ecommandations diales et disponibilité sur le marché	Adoption nationale de la politique	Intégration & préparation du système	Mise en œuvre et offre de service	Disponibilité	Couvertu
ecommandation	**2.1** AMM au niveau national	**3.1** Specifications techniques pour l' acquisition	**4.1** Protocoles partagés au niveau infranational, déconcentré	**5.1** Disponibilité de l'intervention assurée **HHFA 2021**: CaSupp: 24.7%	**6.1** Changement de comportement des ci
rtenariat mondial	**2.2** Adoption/revision de la politique pour l'intervention	**3.2** SIGL du pays	**4.2** Protocoles partagés au niveau des services de soins	**5.2** Aides mémoires disponibles dans les formations sanitaires	**6.2** Couverture de la population assurée
bricants approuvés SRA	**2.3** Guides clinique	**3.3** Indicateurs cibles Intégrés au SNIS	**4.3** Formation des agents de santé		**6.3** Couverture effecti assurée
us les indicateurs eints	**2.4** Liste des médicaments essentiels	**3.4** Curricula de formation mis à jour	**4.4** Supervision, mentorat de routine		**6.4** couverture Equita assurée
elques indicateurs eints / s d'indicateur atteint	**2.5** Engagement des parties prenantes	**3.5** Formation continue mise à jour	**4.5** Activités pour susciter la demande		
ormation non ponible	**2.6** Plan d'action budgétisé				
n applicable pour ervention	**2.7** Ligne budgetaire				

*En utilisant la méthodologie de la revue de la littérature et des enquêtes nationales de l'évaluation des interventions et suivan entretiens avec les informateurs clés, aucune information ni indicateur n'est disponible pour cette intervention

oom, Photos, & Information sur les coûts

f (UNICEF Supply
Calcium Carbonate
ets,100 tablets /

ottle; US$ 0.0225 /

m
$8 pour 30
500 mg,
ns une pharmacie

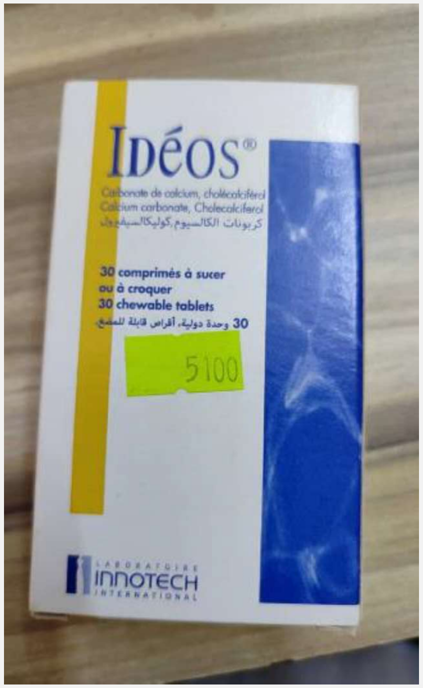

IRSS conduit un essai Clinique randomise Phase III (ECR) pour évaluer l'impact d'une intervention intégrée d'une prise quotidienne CAMMS comparée au fer-acide folique (FAF) pendant la grossesse pour éviter les naissances prématurées et autres problèmes de l'issue de la grossesse

- Toutes les interventions seront dispensées à travers la plateforme de la CPN: Burkina Faso, Pakistan, and Zimbabwe.
- Pour les femmes Burkinabè, mises aléatoirement sous CAMMS, l'aspirine ne sera pas administrée avant la 13eme semaine de grossesse quand il sera possible de l'initier concomitamment avec la sulfadoxine-pyrimethamine.
- Les femmes mises au hasard sous CAMMS recevront chaque semaine des blisters contenant tous les 3 composants: un comprimé de 81 mg d'aspirine, une préparation de Multiple Micronutriments prénataux Internationale des Nations Unies (UNIMMAP) suppléments de multiple micronutriment (MMS), et trois comprimés de 500-mg d'éléments calcium.

See: https://clinicaltrials.gov/study/NCT05612984

PA

ecommandations pour le pays pour la mise à l'echelle

ation d'évidences et dialogue national sont essentiels pour la mise en œuvre de cette intervention

ctions à court terme

- otenir des données récentes sur le statut en calcium des mmes enceintes dans le pays et partager largement les sultats
- upporter une discussion nationale sur l'intégration de la pplémentation en calcium dans les politiques et protocoles
- ur la base de cette décision, mettre à jour les protocoles pour PE/E pour mettre l'accent sur l'utilisation du calcium pour la évention de la prééclampsie
- scrire sur la liste de médicaments essentiels avec l'indication prévention de la prééclampsie
- es données de l' étude sur le calcium, l'aspirine faiblement osée et les multiples micronutriments (CAMMS) pourraient veiller l'engagement pour la mise en œuvre

Actions à moyen terme

- Mettre à jour les outils pour la formation init continue pour intégrer l'utilisation de cette intervention pour la prévention de la préécla au cours de la grossesse
- Travailler avec les bailleurs de fonds et les partenaires de mise en œuvre pour dévelop outils de formation et des aides mémoires
- Réaliser des formations dans tout le pays a l'attention des agents de santé dans les for sanitaires et au niveau communautaire
- Estimer les besoins en calcium pour la prév de la PE/E et l'inclure dans la chaine d'approvisionnement

PA

Aspirine à faible dose (Afd)

FINITION DE L'INTERVENTION: Prise quotidienne d'acide acétylsalicylique à faible
se (75-81 mg). La prophylaxie commence à la fin du premier trimestre pour les
nmes enceintes ayant un risque modéré, à élevé prééclampsie..

acteurs favorisant le passage à l’échelle

ment de politique national mis en place et aspirine à faible dose disponible à un prix dable dans le secteur prive

Aspirine inclus sur la liste des médicaments essentiels LNME comme médicaments non-opioïde, analgésique, antipyrétique et anti-inflammatoire non-stéroïdien, et anti agrégant plaquettaire

Aspirine à faible dose inscrit dans les protocoles nationaux, Protocoles de Santé de la reproduction, Santé de la femme et du nouveau-né de moins de 7 jours (2018) as:

- Aspirine (125 mg) ou 1/4 de 500 mg comprimé pour les femmes enceintes avec un risque élevé de prééclampsie à débuter avec le deuxième trimestre (14ème semaine) jusqu’à la 36ème semaine; prescription basée seulement sur les antécédents maternels et la mesure de la tension artérielle

Aspirine à faible dose est mis en œuvre au niveau des hôpitaux par les gynécologues et dans les formations sanitaires par les sage-femmes et maïeuticiens

Aspirine (500 mg comprimés) est déjà disponible en vente dans les pharmacies privées à un prix abordable

“Les recherche
montré l’importar
l’aspirine à faible
pour la préventio
prééclampsie, et c
enseigné dans
formations .

Agence de plaid
Burkina Fas

BARRIERES AU PASSAGE A L'ECHELLE

nforcer la mise en œuvre en intégrant l'utilisation de l'aspirine à faible dose ns les curricula de formation initiale et continue

La précision de l'âge gestationnel (AG) est limitée; l'échographie n'est pas utilisée habituellement en raison de absence d'échographes

En 2021, environ 40% des femmes ont fait une CPN au cours du premier trimestre

Les femmes n'ont pas d'information sur leurs antécédents sanitaires et il n'y a pas de registre médical électronique, rendant difficile l' évaluation du risque maternel sur la base de son histoire

L'aspirine à faible dose n'est pas inclue dans les modules de formation initiale et continue pour les soins obstétricaux et néonatals d'urgence (SONU)

L'intervention n'est pas suivie dans le système d'information sanitaire (SNIS)

Il n'y a pas d'aide-mémoire

L'aspirine à faible dose n'est pas disponible à la CAMEG, obligeant les femmes dans le besoin à l'acheter dans le secteur privé au lieu de le recevoir gratuitement dans les services de santé dans le cadre de la gratuité. Cela empêche les plus vulnérables d'avoir accès à l'aspirine à faible dose.

Aspirine 500 mg comprimé est distribué dans le secteur public à travers la CAMEG pour de multiples indications et les ruptures sont fréquentes

"Insufficience da
communication av
femmes, à qui o
donne pas d'inform
sur leurs maladies
et l'importance d
signaler lors des f
grossesses."

Agence de plaid
Burkina Faso

ogrès vers les étapes essentielles du passage à l’ échelle

d’indicateurs direct; les données de disponibilité ne sont pas spécifique à l’aspirine à faible dose

ecommandations diales et disponibilité sur le marché	Adoption nationale de la politique	Intégration & préparation du système	Mise en œuvre et offre de service	Disponibilité	Couvertu
ecommandation	**2.1** AMM au niveau national	**3.1** Specifications techniques pour l’ acquisition	**4.1** Protocoles partagés au niveau infranational, déconcentré	**5.1** Disponibilité de l’intervention assurée **SARA 2018: 31% aspirin (dose unknown) available**	**6.1** Changement de comportement des cibles
artenariat mondial	**2.2** Adoption/revision de la politique pour l'intervention	**3.2** SIGL du pays	**4.2** Protocoles partagés au niveau des services de soins		**6.2** Couverture de la population assurée
abricants uvés OMS/SRA	**2.3** Guides clinique	**3.3** Indicateurs cibles Intégrés au SNIS	**4.3** Formation des agents de santé	**5.2** Aides mémoires disponibles dans les formations sanitaires	**6.3** Couverture effe assurée
	**2.4** Liste des médicaments essentiels	**3.4** Curricula de formation mis à jour	**4.4** Supervision, mentorat de routine		**6.4** couverture Equi assurée
	**2.5** Engagement des parties prenantes	**3.5** Formation continue mise à jour	**4.5** Activités pour susciter la demande		
	**2.6** Plan d’action budgétisé				
	**2.7** Ligne budgetaire				

Légende:
- us les indicateurs eints
- elques indicateurs eints
- s d’indicateur atteint
- ormation non ponible
- n applicable pour ervention

*En utilisant la méthodologie de la revue de la littérature et des enquêtes nationales de l’évaluation des interventions et suivan entretiens avec les informateurs clés, aucune information ni indicateur n’est disponible pour cette intervention

oom, Photos, & Information sur les coûts

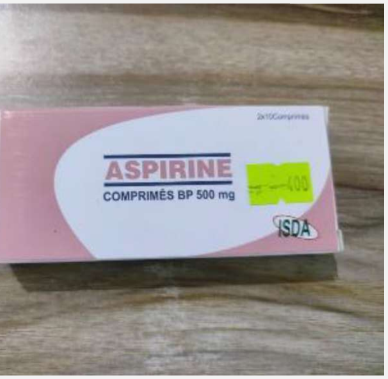

t: Acide
alicylique 500 mg
més, blister de 10 Boite
omprimés (non-
e)
dicatif (UNICEF):
32 / blister
0057 / comprimé
12 tout le traitement

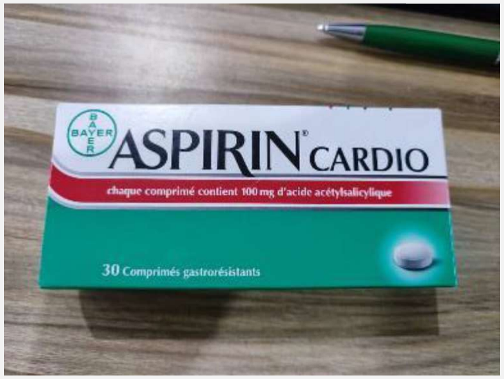

Produit: Aspirine a faible dose
CFA 1800/US$2.94 pour 30 comprimés de100 mg (vendu avec une indication de cardiovasculaire); prix obtenu dans une pharmacie privée

IRSS conduit ECR phase III pour évaluer l'impact d'une combinaison quotidienne de CAMMS comparée au fer acide folique pendant la grossesse sur les accouchements prématurés et les autres mauvaises issues de la grossesse à travers la plateforme de la CPN existante dans 3 pays : Burkina Faso, Pakistan et Zimbabwe.

- Pour les femmes Burkinabè, mises aléatoirement sous CAMMS, l'aspirine ne sera pas administrée avant la 13ème semaine de grossesse quand il sera possible de l'initier concomitamment avec la sulfadoxine-pyrimethamine.
- Les femmes mises au hasard sous CAMMS recevront chaque semaine des blisters contenant tous les 3 composants: un comprimé de 81 mg d'aspirine, une préparation de Multiple Micronutriments prénataux Internationale des Nations Unies (UNIMMAP) suppléments de multiples micronutriments (MMS), et trois comprimés de 500-mg d'éléments calcium.
- Voire : https://clinicaltrials.gov/study/NCT05612984

UNFPA supporte la mise en œuvre d'un projet de renforcement de la disponibilité des échographes et de formation des sage-femmes à l'utilisation dans le cadre de la mise en œuvre de la recommandation de l'OMS pour les 8 contacts prénatals au cours de la grossesse

ecommandations pour le pays pour la mise à l’echelle

compagner l’élan de la direction de la santé de la famille (DSF) pour réviser les modules SONU afin d’y inclure révention de la prééclampsie par l’utilisation de l’aspirine à faible dose

Actions à court terme

Continuer à encourager à fréquenter la consultation prénatale, plus particulièrement au premier trimestre et renforcer la communication/conseils autour de la maladie hypertensive de la grossesse.

Inscrire l’aspirine à faible dose sur la liste nationale des médicaments essentiels LNME avec l’ indication pour PE

Prioriser l’inclusion l’aspirine à faible dose dans les formations initiales et continue [modules SONU]

Travailler avec les bailleurs et les agences de mise en œuvre pour élaborer des modules de formation et des aide-mémoires

Encourager la CAMEG à conduire une quantification pour l’aspirine à faible dose pour la prévention de la PE en collaboration avec la MSPH

Actions à moyen terme

- Conduire des formations des agents de santé au plan national tant au niveau des formations sanitaires qu’au niveau communautaire
- Intégrer la distribution de l’aspirine à faible dose dans la chaine d’approvisionnement d secteur publique via la CAMEG
- Intégrer les indicateurs pertinents au système d’information sanitaire pour suivre l’utilisation de l’aspirine à faible dose

Ethiopia

Methods and data sources

ethods for key informant interviews (KII) and analysis

A structured interview guide was used to guide the discussion, organized along the stages of scale up framework

Virtual and in-person consultations were conducted from June–August 2023 with relevant organizational representatives

Respondents provided verbal consent to participate

Handwritten and electronic notes were expanded, entered in a Smartsheet form, and exported into a framework to support cross-respondent analysis by key themes

Some participants shared additional programmatic reports, grey literature, and other document resources following the KII

ummary of KIIs

Total number of KIIs conducted (n=17), and by type

- ❖ Implementers = 5
- ❖ Advocates = 8
- ❖ Decisionmakers = 4

List of organizations consulted

- Ministry of Health (MOH) Maternal and Chil
- MOH Nutrition
- MOH Medical Equipment and Medicine Sup
- Ethiopia Pharmaceutical Supply Service (EF
- Jhpiego
- Clinton Health Access Initiative (CHAI)
- Harvard Haset Project
- Ethiopian Midwives Association (EMwA)
- Ethiopian Pediatrics Society (EPS)
- Ethiopian Society of Obstetricians and Gynecologists (ESOG)
- World Health Organization (WHO)
- Global Financing Facility (GFF)

scription of data sources reviewed

e of Sources	Total sources (n=48) reviewed across all 8 assets
sites	5 WHO, MOH, EFDA, EPSS, Unicef Supply Catalogue
pal Guidelines	7 global reports and guidelines
onal Market<br>horization<br>uments	2 sources, including eIRS EFDA (2023); List of medicines for community pharmacy, EFMHACA/EFDA (2012).
onal Policy<br>uments	4 national policies including HSTP II | 2020/21-2024/25; National Strategy for Newborn and Child Survival in Ethiopia 2015/16-2019/20; National strategy for Newborn and Child Health and Development (2022); Reproductive Health (RH) Strategic Plan M (2021–2025).
onal Reports	3 including Annual Report of 2013 EFY; Special Bulletin 22nd annual review meeting MOH 2020; MOH annual performance rep (2020/21)
onal Clinical<br>lelines & Protocols	9 guidelines, including ANC Guideline 2021; Kangaroo Mother Care Technical and Implementation Guideline 2023; Neonatal Intensive Care Unit (NICU) Implementation Guide 2016; NICU Management Protocol; Obstetrics Management Protocol for Hea Centers MOH, Ethiopia, 2021; Obstetrics Management Protocol for Hospitals. MOH, Ethiopia, 2021; PPH National Guidelines (IMNCI guidelines; ICCM guidelines.
ential Medicine<br>s (EML)	3 EMLs, including Ethiopia's EML (2020); National Medical Instruments List with minimum specification (2013); Pharmaceutica procurement list, EPSA, 2018
S / Stockout<br>rts	4 including HMIS indicator reference sheet; DHIS2; NICU register; Maternal and perinatal death surveillance and response (MF system
onal Surveys	6 surveys: PMA Ethiopia Unique Survey report 2020; SARA 2018; SPA 2022; Demographic and Health Survey 2016; Survey o Micronutrient by EPHI in 2016; National Food and Nutrient Strategy Baseline Survey 2023.
ning Curriculums	5 including, Oxygen Therapy in Children and Adults Participant's Manual 2017; ICCM for HEWs 2018; BEmOC; IMNCI; Mento Guideline for Reproductive, Maternal and Newborn Health 2017

Results

hiopia country context

lity of maternal newborn care	Indicator Value E-DHS 2019
ng of first antenatal check: less n 4 months	27.8%
lian months of pregnancy at first enatal care visit	4.5
enatal visits for pregnancy: 1	3.3%
enatal visits for pregnancy: 4+	43.0%
istance during delivery from a ed provider	49.8%

Average proportion of Essential drugs available in Health Facilities (SARA 2018)	Indicator Value
PHC/health center	48%
Secondary/referral hospital	86%
All/public health facilities	49%
Note: The proportion were determined through computation of essential drugs list	

ilability of Essential Drugs and Guidelines

ential drugs were available on average in 78% of the hospitals (referral, general, and primary hospitals) and 57% health centers/PHC (E-SPA, 2021-22).

C guidelines or other guidelines relevant to ANC were available in 52% of facilities that offer ANC services. The delines are more likely to be available in hospitals and health centers than in the health post (ESPA, 2021/22).

Calcium Supplementation (CaSupp)

SET DEFINITION: In areas with low dietary calcium intake, pregnant women take 1.5–2.0 g of mental calcium daily spread over three doses per day for the prevention of pre-eclampsia.

NABLERS TO SCALE

pportive policy and clinical guidelines environment, wide availability, and high ognition of importance of CaSupp among national level stakeholders enable scale

portive policy and clinical guidelines environment

aSupp for PE/E prevention included in the Reproductive Health Strategy (2021-025), ANC guidelines (2022), Obstetric Management Protocols (2021), and harmaceuticals Procurement List (2018)

n-service and pre-service trainings updated for CaSupp (e.g., for ANC and BEmOC)

ket authorization and procurement

he nEML lists calcium carbonate (600 mg tablet) and calcium gluconate (500 mg ablet) for correcting fluid, electrolyte, and acid base balance; **however, there is no ndication for prevention of pre-eclampsia.**

he list of medicines for community pharmacy, EFMHACA/EFDA (2012), includes alcium carbonate (Tablet, 350 mg, 500 mg, 700 mg) for antacids under astrointestinal medicines; 350 mg tablet is a tracer product for the revolving drug und (RDF) essential drug list.

n-country market authorization for five CaSupp products, although none are locally nanufactured (eRIS, EFDA 2023) (see notes section for full list)

"So far, we haven implementation o supplementation endorsing the documents [guidelines and protocols], beca financial short

Decision-maker,

NABLERS TO SCALE

pportive policy and clinical guidelines environment, wide availability, and high ognition of importance of CaSupp among national level stakeholders enable scale

keholder engagement

ctive engagement of stakeholders/partners to support the scale up, including ational level advocacy promoting CaSupp; for example, during the national PE/E ay celebrated for 2nd time on May 22, 2023

al data on calcium intake

wo recent surveys determined dietary calcium intake among women of eproductive age is up to 70% less than the recommended amount; additional tudies show women consume insufficient calcium (Tesfaye et al:2018) and a ignificant association between low dietary calcium intake and low erum calcium levels in women with pre-eclampsia (Gebreyohannes et al:2021)

"Calcium supplem
is a proven interv
prevent about 7
preeclampsia, w
the second cau
maternal dea
Ethiopia. There
advocacy is critic
step for the sca

Decision-maker,

BARRIERS TO SCALE

ack of ANC provider awareness, low and late ANC coverage, and high commodity osts impedes the use of CaSupp

Late and low ANC coverage: Only 28% of women had their first ANC visit during the 1st trimester; and less than half (43%) of women had at least four ANC visits during their last pregnancy (Mini EDHS 2019)

Low awareness of CaSupp among service providers and no inclusion of CaSupp within ANC-related job aids. Only 7% of providers surveyed reported in-service training on ANC in the past 6 months, while 23% reported this ever (ESPA 2021/22)

Cost effectiveness: One modeling shows CaSupp for PE/E prevention in Ethiopia was not cost-effective, with a cost per DALY of about US$3,100 (Memirie et al:2019), but MOH and other key stakeholders believe the intervention is cost effective.

Dosing: Simulation models show that providing CaSupp of 1.5–2.0 g/d could result in high proportions of women exceeding the Upper Intake Level (Tesfaye et al:2018)

Procurement: CaSupp not included in MOH lifesaving maternal health commodity list

Availability and coverage data: CaSupp not included in HMIS/DHIS2/LMIS

Calcium can inhibit iron absorption. Ethiopia's ANC guidelines state the two nutrients should preferably be administered >3 hours apart.

> "Iron and calci
> supplementation ar
> nutrition progr
> commodities…the co
> alone was quite hig
> MoH, and on top of t
> another supplement
> for ANC would incre
> nutrition program co
> challenging.
>
> *Decision-maker, E*

rogress toward milestones

ny national policy adoption indicators and training updates have been met, but progress is needed on monitoring the availability a
erage of calcium supplementation among pregnant women.

obal guidelines & arket availability	National policy adoption	System integration & readiness	Implementation & service delivery	Availability	Coverage
HO Guidelines	**2.1** National SRA/ Regulatory Body Approval	**3.1** Commodity Procurement Specifications	**4.1** Guidelines Disseminated to Subnational-Level	**5.1** Asset Availability Ensured	**6.1** Behavior Change of Audience Members
obal Partners	**2.2** Policy Adopted/ Revised for Asset	**3.2** Country LMIS	**4.2** Guidelines Disseminated to Service-Delivery Level	**5.2** Job Aids Available at Facility Level	**6.2** Population Coverage Achieved
HO/SRA ated acturers	**2.3** Clinical Guidelines	**3.3** Relevant Indicators Integrated into HMIS	**4.3** HCW Implementation Training		**6.3** Effective Coverage Achieved
	**2.4** Essential Medicines List	**3.4** Training Curricula Updated	**4.4** Routine Mentorship		**6.4** Equitable Coverage Achieved
	**2.5** Stakeholders Engaged	**3.5** In-service Training Updated	**4.5** Demand Generation Activities		
	**2.6** Costed Implementation Plan				
	**2.7** Budget Line				

ndicators met

ne indicators met

indicators met

rmation
vailable*

applicable for
asset

*Using the Asset Tracker methodology of conducting literature review of policies and national surveys, and following up with key informant interviews, no information was available for this asset and indicator.

otlight, Photos, & Cost Information

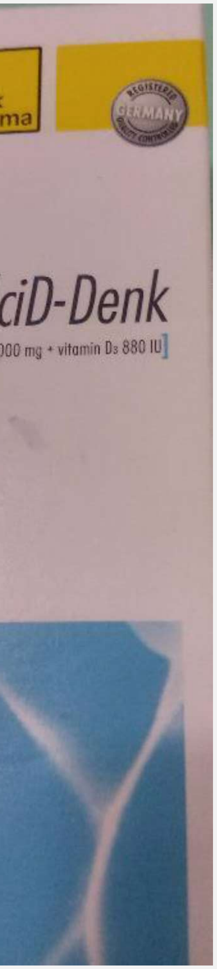

Product: CalciD-Denk in a package of 20 tablets, each tablet containing 1,000 mg calcium carbonate and 22 micro grams vitamin D3.

Price: 900 Birr / US$ 16.00 (20 tablets); US$ 0.80 per tablet

Source: Obtained from a private pharmacy in Addis Ababa

Indicative Pricing (UNICEF supply catalog, 2023):
Calcium Carbonate 1500mg tablets, 100 tablets/bottle.
US$ 2.25 / bottle; US$ 0.0225 / tablet

SPOTLIGHT

A study in Ethiopia showed significant association between low dietary calcium intake and low serum calcium levels with pre-eclampsia.

This local evidence supports current context-specific recommendation of calcium supplementation in societies with low-dietary calcium consumption to prevent pre-eclampsia.

An implementation study should be considered in Ethiopia to assess feasibility of routine supplementation. (Gebreyohannes et al., 2021).

eliminary recommendations for the country to advance scale up

areness raising of CaSupp among providers to conform to ANC guidelines and quantification and budget cation are critical actions.

term actions (0-2 years)

- ain service providers and ensure efficient use by ly providing CaSupp to the targeted high-risk groups
- rgeted and evidence-based advocacy to win cision-makers and stakeholders' support financing CaSupp
- sure forecasting, costing, and budget allocation for Supp
- alyze health facility reports (and other data sources) increasing maternal deaths from hypertension and e evidence to inform decision-making around Supp scale up
- sure updating EML to list CaSupp (1.5–2.0 g of emental calcium daily spread over three doses per y) for the prevention of pre-eclampsia.

Medium-term actions (2-5 years)

- Update the MOH priority maternal health commodity include CaSupp
- Given limited resources, consider implementation of based geographic risk stratification
- Include CaSupp indicators into HMIS, LMIS, and oth surveys (e.g., EDHS, SPA, SARA etc.) to assess ad and monitor service delivery and measure coverage
- Consider different, more feasible dosage of calcium (500mg–1g) to reach more women
- Streamline decisions around recommended formulat ANC distribution
- Consider implementation study in Ethiopia to look fo feasibility of routine supplementation

ow-dose Aspirin (LDA)

SET DEFINITION: Daily low-dose aspirin (75-81mg) prophylaxis beginning in the late first trimester
pregnant women at moderate to high risk of pre-eclampsia.

NABLERS TO SCALE

pportive policy environment and wide availability and access to affordable LDA enable scale

pportive and clinical guidelines policy environment

ANC Guidelines (2022) recommend LDA for prevention of PE in women at high risk of developing PE. Risk assessed by maternal history, blood pressure, and risk assessment based on risk factors (first pregnancy, age <18 or >35, multiple gestation, history of hypertension, renal disease, diabetes, obesity, or family history of PE); pregnant women at high risk referred to hospital for initiation of aspirin.

Obstetric Management Protocol for Hospitals (2021) states LDA as a preventive medication after 12 weeks' gestation.

n-service training curricula updated to include LDA

Acetylsalicylic Acid Tablet (75 mg, 81 mg, 100 mg, 300 mg) listed on nEML for anti-platelet under "Cardiovascular Medicines". **There is no specification of low dose aspirin for PE/E prevention.**

MOH is working to develop a protocol on pre-eclampsia and eclampsia management hat will be published soon (still in progress).

"At the national le
government
recognized the im
of low dose aspiri
supported or refl
policy docume
example low dose
is included in the
antenatal care gu

Decision-maker,

NABLERS TO SCALE

pportive policy environment and wide availability and access to affordable LDA enable scale

keholder engagement

xisting national maternal health TWG engaged around LDA

rket authorization and procurement

LDA products (81 and 100 mg) received market authorization, including two locally manufactured products:

- Aspi-SSP 81: 81mg tablets,10 tablet strip (Sansheng Pharmaceutical, Ethiopia)
- E-SPIRINS: 81mg tablets, 10 tablet blister (Ethiopian Pharmaceutical Manufacturing, Ethiopia)
- Aspirin Cardio: 100mg tablets, 3 blister packs of 10 tablets (Bayer AG, Germany)

LDA (81 mg) is included in the MOH priority list of lifesaving maternal health commodities, included in the EPSS pharmaceuticals procurement list, and regularly monitored within the RDF to assess EPSS performance.

LDA (81mg) budgeted in national costed implementation plan with facilities expected to budget and procure LDA through EPSS via the RDF.

Wide availability of LDA (81 mg) in the private and public sectors at an affordable price

"Procurement of
government bu
started two years
so far, there is n
with the availab
81mg LDA. We s
interventions with
LDA but now the
is updated fo
75mg LDA. Acco
need to upda
procurement spe
to 75mg LD

Decision-maker

BARRIERS TO SCALE

ack of ANC provider awareness and low and late ANC coverage impedes use of LDA

Late and low antenatal coverage for early risk identification: only 28% of women had their 1st ANC visit during the 1st trimester; and less than half (43%) of women had ≥ four ANC visits during their last pregnancy (Mini EDHS 2019)

Limited awareness about LDA among ANC providers at the lower levels; and **no job aid for LDA**

Difficulty in determining the GA for risk assessment due to lack of ultrasound at lower levels of care and memory lapse of mothers regarding last menstrual period

Lack of evidence base for implementation of LDA in Ethiopia (Gudu & Sripod:2020)

Perceived risk of confusion between low- and high-dose product; use of high-dose aspirin (300 mg) can increase risk of hemorrhage in pregnant women

Need for referral and transport to hospital for initiation of LDA in high-risk women

Procurement: LDA not included in MOH lifesaving maternal health commodity list

Availability and coverage data: LDA not included in HMIS/DHIS-II/LMIS

Adherence: LDA and other self-administered interventions (CaSupp, IFA, MMS…) are recommended at the same time which could affect the adherence

"The major side effe
dose] aspirin is her
because it interferes
clotting…In most c
pharmacies properly
between high and
aspirin and dispense
but still we cannot e
risk of confusion…
building of health wo
drug dispensers esp
periphery level sh
supported by job aids
help reduce the
confusion.

Decision-ma
Ethiopia

rogress toward milestones

ny national policy adoption indicators and training updates have been met, and LDA is widely available at a low cost; however, tra
erage of LDA among pregnant women is needed for monitoring scale up.

bal guidelines & arket availability	National policy adoption	System integration & readiness	Implementation & service delivery	Availability	Coverage
HO Guidelines	**2.1** National SRA/ Regulatory Body Approval	**3.1** Commodity Procurement Specifications	**4.1** Guidelines Disseminated to Subnational-Level	**5.1** Asset Availability Ensured	**6.1** Behavior Change of Audience Members
bal Partners	**2.2** Policy Adopted/ Revised for Asset	**3.2** Country LMIS	**4.2** Guidelines Disseminated to Service-Delivery Level	**5.2** Job Aids Available at Facility Level	**6.2** Population Coverage Achieved
HO/SRA ated acturers	**2.3** Clinical Guidelines	**3.3** Relevant Indicators Integrated into HMIS	**4.3** HCW Implementation Training		**6.3** Effective Coverage Achieved
	**2.4** Essential Medicines List	**3.4** Training Curricula Updated	**4.4** Routine Mentorship		**6.4** Equitable Coverage Achieved
	**2.5** Stakeholders Engaged	**3.5** In-service Training Updated	**4.5** Demand Generation Activities		
	**2.6** Costed Implementation Plan				
	**2.7** Budget Line				

ndicators met

ne indicators met

indicators met

rmation
vailable*

applicable for
asset

*Using the Asset Tracker methodology of conducting literature review of policies and national surveys, and following up with key informant interviews, no information was available for this asset and indicator.

otlight, Photos & Cost Information

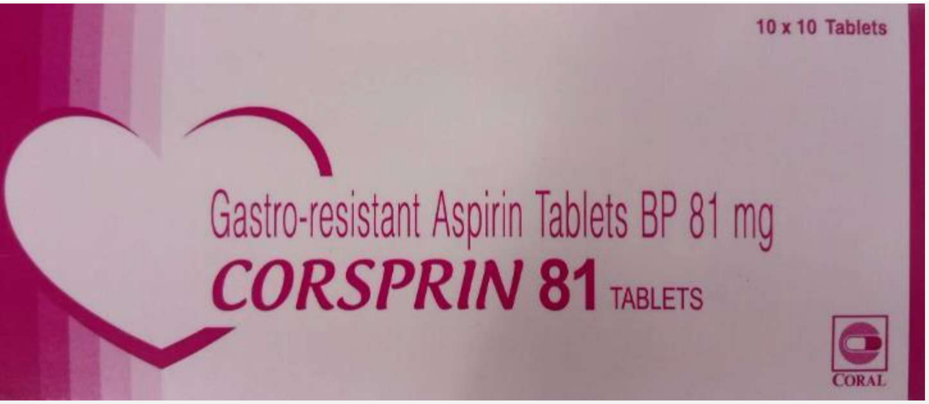

luct: Corsprin 81 mg (100 tablets)

e: 69 Birr / US$ 1.09 / pack; US$ 0.011 / tablet

rce: obtained from private pharmacy in Addis Ababa

checks at 16 private pharmacies in Addis: 11 macists reported previously stocking the 75mg ulation, but it expired a few months ago (primarily ensed for cardiac cases, but demand was low), and armacists reported never dispensing 75mg LDA.

cative Pricing (from UNICEF supply catalog, 2023): ylsalicylic acid 75mg tablets, blister pack of 56 tablets. 0.32 / blister pack; US$ 0.0057 / tablet

SPOTLIGHT

Senior professionals from the associations like Ethiopian Society of Obstetricians / Gynecologists (ESOG) and Ethiopian Midwives Association (EMwA) were engaged in advocacy to ensure inclusion of LDA in policy and guidelines.

"There was strong engagement of professional associations and senior professionals from the associations like ESOG, and EMwA were in the advocacy to ensure inclusion of LDA in policy and guidelines."

Decision-maker, Ethiopia

eliminary recommendations for the country to advance scale up

ude commodity procurement of LDA for women at risk of PE and train providers in use of LDA product

t-term actions (0-2 years)

nsure ANC guidelines (2022) are provided to all health facilities

uild capacity of the ANC service providers through training and entorship, especially at PHC level about LDA use

evelop and avail job aid to service providers

nsure the 2024 EML revision by EFDA lists LDA (75-81mg) for ophylaxis for pregnant women at moderate to high risk of PE

dvocacy to win the support of the national and subnational akeholders in providing technical and financial support for the scale up

nsure quantification and allocation of budget for LDA for women at risk PE/E

rengthen demand creation for ANC to increase early care seeking, ssibly by engaging religious/traditional healers in shifting community vareness of PE/E (Robins et al:2021)

Medium-term actions (2-3 year

- Scale up ultrasound service (to h centers) and build capacity for se providers on ultrasound
- Include LDA use indicators into H LMIS, and other relevant surveys EDHS, SPA, SARA etc.) to asse adherence and monitor service delivery and measure coverage

Kenya

Methods and data sources

lethods for key informant nterviews and analysis

A structured interview guide was used to guide the discussion, organized along the stages of scale up framework

Virtual and in-person consultations were conducted from August - October 2023 with relevant organizational representatives

Respondents provided verbal consent to participate

Handwritten and electronic notes were expanded following the interview, then transcribed electronically and entered in a Smartsheet form, and exported into a framework to support cross-respondent analysis by key themes

Some organizations shared additional programmatic reports, grey literature, and other document resources following the interview

List of organizations consultec

- MOH
- The University of Nairobi
- UNFPA
- USAID
- UNICEF
- Kakamega MNCH CSO Alliance
- Medicines Transparency Alliance (MeTA)
- Kilifi County government
- Nutrition International
- Nest 360

ata sources summary

Total number of source documents reviewed: 40

Note this total is across all assets, including policies, guidelines, EMLs, etc., any documents we are using as sources for information in indicator mapping

Total number of KIIs conducted (n=16), and by type:

- Implementers = 8
- Advocates = 3
- Decision-makers = 5

ata sources summary

ype of Sources	Total Sources Reviewed across all 8 assets = 46
/ebsites	**4** (1 global coalition, DefeatDD; 2 for in-country TWGs; Kenya Pharmacy and Poisons Board)
lobal Guidelines ocuments	**16** documents (from WHO, FIGO, ACOG, Unicef, and Nutrition International)
larket Authorization ocuments	**1** documents (KEMSA Tender Documentation)
ational Policy ocument(s)	**3** documents (National Nutrition Action Plans, National MIYCN Policy, National Guidelines for Quality Obstetrics and Perinatal Care)
ational Clinical uidelines & Protocols	**4** documents (Kenya Basic Paediatric Protocols; Standards for Maternal Care; Guideline on manageme apnoea of prematurity; Comprehensive Newborn Care Protocols)
MLs	**4** EMLs (KEML 2023, KEML 2019, KEML 2016, WHO EML 2021)
MIS	**1** database (Kenya DHIS2 to review indicators)
ational Surveys	**4** surveys (KDHS 2022, KHFA 2018/19, SARA 2013, SPA 2010)
lobal Surveys	**2** surveys (MCGL 2023, WHO SRMNCAH 2018/19)
raining Curriculums	**7** documents (EmONC mentor and mentee manuals; IMNCI booklet, facilitator and participant manuals; facilitators manual; Newborn Care ETAT training package for pre-service and in-service).

Results

ya country context

lity of maternal newborn care	Value	Source
ng of first antenatal check: less than 4 months	28.6%	KDHS 2022
ian months of pregnancy at first antenatal care visit	4.9m	KDHS 2022
natal visits for pregnancy: 1	2.7%	KDHS 2022
natal visits for pregnancy: 4+	66.0%	KDHS 2022
natal visits for pregnancy: 8+ visits	4.0%	KDHS 2022
stance during delivery from a skilled provider	89.3%	KDHS 2022
**nmodity availability**	**Value**	**Source**
rin (dose unknown) capsule/tablet available	50% -61%	KHFA 2018/19
natal corticosteroids available	45%	KHFA 2018/19
lytics (Nifedipine) available	80%	Momentum 2022
romycin	80%	KHFA 2015
rehydration solution available	82%	KHFA 2018/19
sulphate tablet or syrup	81%	KHFA 2018/19

PA

alcium Supplementation
CaSupp)

SET DEFINITION: In areas with low dietary calcium intake, pregnant women take 1.5–2.0 g of mental calcium daily spread over three doses per day for the prevention of pre-eclampsia.

OVERVIEW:
CIUM SUPPLEMENTATION upp)

Complicated regimen and conditional global recommendations leaves countries without actionable plans aligning with optimal counseling models for improved adherence; CaSupp is accepte not prescribed.

kground & context

inition: Calcium entation during pregnancy as in areas with low lcium intake, pregnant ke 1.5-2.0 g of elemental aily spread over three r day for the prevention of npsia[1]

ost recent calcium entation during pregnancy was released in 2018

l Guidelines for entation pre-pregnancy n 2020, recommends in research contexts only

obal survey of 31 studying prevention of sive disorders identified 6 untries surveyed included upplementation l guidelines, and 71% nclusion in public pre- nd in-service training.[2]

2 Scale-up status

Global guidelines & market availability (A) → National policy adoption (B) → System integration & readiness → Implementation & service delivery (C) → Availability → Cove

3 Common barriers and enablers to scale

A WHO recommended regimen challenging to meet

Current dosage regimen perceived as high. Current research investigating possibility of impact at lower supplementation levels to reduce required dosage. Latest evidence indicates noninferiority of lower dosage[3]

High dosage leads to logistical challenges Distribution of 3 tablets per day of bulky supplement is logistically challenging without concerted effort and planning

Deviation from WHO in national guidelines Kenya, for example, includes 1.0g/day as the guideline, lower than current recommendation[4]

B Complicated and unclear implementation recommendation

Conditional recommendation focused on low dietary intake of calcium confusing for decision-makers. When dietary information is available, there is limited information on calcium intake, rarely stratified by pregnant women. Global known dietary calcium intake in LMICs is typically lower than recommendations[3]

Definition of low dietary intake is not universal. Different governing bodies prescribe varying levels of optimal dietary calcium intake during pregnancy, typically ranging from 900–1200 mg/day

> *"The easier one makes it [to implement, to take], the more likely it will be used."*
>
> Global level, Advocate

C Product cost and size barriers to implement

IFA or MMS supplementatio current priority and cheaper, calcium is seen as a cost-bar already challenging ANC imp

Calcium supplements are b creating perceived challeng women with the quantity re Supply chain difficulties for st forecasting supply needs, an

NABLERS TO SCALE

Supp is included in the national guidelines and evidence on CaSupp integration conducted

upp for prevention of PE/E is recommended in the National Guidelines on Quality
tetrics and Perinatal Care (2022):

- For prevention of PE and it's complications, high risk women take calcium supplementation of at least 1g/day if calcium dietary intake is low.
- MOH encourages Kenyans to eat at least 5 foods groups among the 10 groups, every day, Pg 35 National Guidelines on Quality Obstetrics and Perinatal Care (2022).

rgency Obstetrics and Newborn care pre-service and in service curricula (EmONC)
2, updated to include CaSupp.and mentors in 38 counties have been trained.

ium Carbonate (1.25 g, 500 mg) included in KEML 2023 for use in Level 4 facilities
ove

ium Carbonate 500 mg indication for prevention and treatment of calcium deficiency
lts 2 to 3 tablets daily) is in the Kenya National Medicine Formulary (KNMF) 2023

ults of a feasibility and acceptability study on integrating iron with CaSupp in pregnancy
ved greater calcium intake for those assigned 3 vs. 2 doses per day. The findings and
mmendations can support introduction and scale up in Kenya (Omotayo et al, 2018)

"Prescription
individualized b
on individua
needs / conditi

Decision ma
Kenya"

BARRIERS TO SCALE

ck of awareness of importance of CaSupp for PE/E and a disjointed approach to roduction at national level

The national guidelines do not align with the WHO guidelines for dosage (guidelines indicate 1g as compared to 1.5–2g in 2018 WHO guidelines).

Though mentors in 38 counties have been trained, the cascading down to facility level has been slow.

There is no national data to show the calcium deficiency burden in the country. The last micronutrient survey in the country did not assess calcium deficiency.

There is different views and focus between the Maternal health and Nutrition space around calcium supplementation during pregnancy.

There is no known costed implementation plan or budget line for CaSupp, and it is not procured routinely.

CaSupp is costly and may not be affordable in the public sector.

> There lacks ev
> at national le
> calcium defici
> pregnant wome
> a public he
> concern
>
> *Decision make*

rogress toward milestones

onal policy adoption indicators and training updates have been met, but progress is needed on monitoring the availability and cov
lcium supplementation among pregnant women.

obal guidelines & arket availability	National policy adoption	System integration & readiness	Implementation & service delivery	Availability	Coverage
HO Guidelines	**2.1** National SRA/ Regulatory Body Approval	**3.1** Commodity Procurement Specifications	**4.1** Guidelines Disseminated to Subnational-Level	**5.1** Asset Availability Ensured	**6.1** Behavior Change of Audience Members
obal Partners	**2.2** Policy Adopted/ Revised for Asset	**3.2** Country LMIS	**4.2** Guidelines Disseminated to Service-Delivery Level	**5.2** Job Aids Available at Facility Level	**6.2** Population Coverage Achieved
HO/SRA ated acturers	**2.3** Clinical Guidelines	**3.3** Relevant Indicators Integrated into HMIS	**4.3** HCW Implementation Training		**6.3** Effective Coverage Achieved
	**2.4** Essential Medicines List	**3.4** Training Curricula Updated	**4.4** Routine Mentorship		**6.4** Equitable Coverage Achieved
	**2.5** Stakeholders Engaged	**3.5** In-service Training Updated	**4.5** Demand Generation Activities		
	**2.6** Costed Implementation Plan				
	**2.7** Budget Line				

ndicators met

ne indicators met

indicators met

rmation vailable*

applicable for asset

*Using the Asset Tracker methodology of conducting literature review of policies and national surveys, and following up with key informant interviews, no information was available for this asset and indicator.

otlight, Photos, & Cost Information

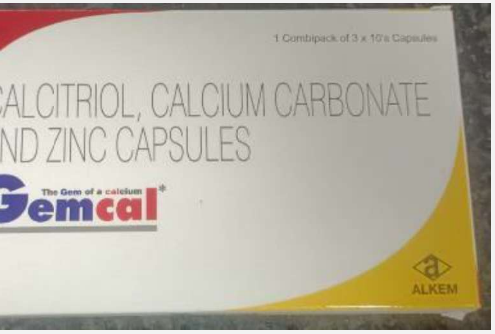

Product: Calcitriol, calcium carbonate and Zinc Capsules (Gemcal)

Pricing:
KES 1650 for 30 tablets (KES 55 per tablet // US$0.40 per tablet)

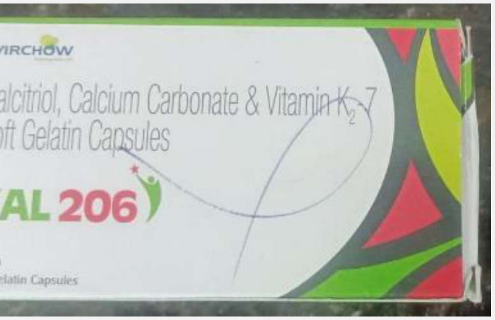

Product: Calcitriol, Calcium carbonate and vitamin K (KAL 206)

Pricing:
KES 1140 for 30 tablets (KES 38 per tablet // US$0.26 per tablet)

cing obtained from a private pharmacy (Source: Janet Shauri)

icative Pricing (UNICEF Supply Catalogue 2023):
cium Carbonate 1500mg tablets,100 tablets / bottle.
$2.25 / bottle; US$0.0225 / tablet

SPOTLIGHT

A study in Western Kenya explored factors that influence adoption and acceptability of calcium supplementation for prevention of pre-eclampsia among pregnant women in rural communities. Findings showed pregnant women are likely to adopt CaSupp with appropriate programmatic adaptations. Careful attention to product attributes, regimen complexity, and strategies for reassuring and reminding women are needed to adapt the WHO guidelines.

(Omotayo et al., 2018)

ecommendations for the country to advance scale up

ving from research, policy alignment then to implementation of CaSupp

mediate near-term actions

Advocacy, policy change, and dialogue to elevate eclampsia as a cause of maternal death, building a case for introduction and scale of CaSupp.

Disseminate and utilize research findings in implementation of CaSupp.

Adopt a multidisciplinary approach to CaSupp; a dedicated team from both the Division of Reproductive and Maternal Health and the Division of Nutrition to examine existing opportunities for CaSupp. Advocacy among key stakeholders is needed to determine best way forward.

For equitable access of CaSupp, invest in market shaping activities (Affordability availability, assured quality, appropriate design, awareness)

Disseminate national guidelines to the counties and facility level.

Initiate HCW training, mentorship and develop job aids to ensure effective utilization.

Update the Emergency Obstetrics and Newborn Care Guidelines and pre-service and in service curricula to incorporate use of CaSupp in preventing pre-eclampsia during pregnancy to align with WHO 2018 recommendations (1.5–2g daily with caution of iron interaction)

Medium-term actions

- Demand generation activities are required to awareness of the indication to drive utilizatior
- Availability of the calcium supplements for PE public procurement agencies (KEMSA and M
- CaSupp should be included in national and c workplans and budgets
- Survey to identify geographical regions with h calcium deficiency in Kenya

PA

Low-dose Aspirin (LDA)

SET DEFINITION: Daily low-dose aspirin prophylaxis beginning in the late first trimester for egnant women at moderate to high risk of pre-eclampsia.

OVERVIEW:

-DOSE ASPIRIN (LDA)

Progress toward scale hampered by variable policy framework, low provider and community awareness, late antenatal care attendance and under resourced health systems.

kground & context

inition: Daily low-dose 5-81mg) prophylaxis for women at moderate to of pre-eclampsia by twenty weeks

21 guidance recommends A (75 mg/day) for t moderate and high risk lding on WHO 2011 ndation that focused only n at high risk.

c since majority of me to ANC after 20 DA should be started t; so, most women missing the boat'. a of introducing LDA pp] early is great, so if do that because e not presenting early ow effective is it?"

Implementer, Global level

2 Scale-up status

Global guidelines & market availability (A) → National policy adoption → System integration & readiness (B) (C) → Implementation & service delivery → Availability → Cover

3 Common barriers and enablers to scale

A Barriers to policy implementation

Socialization of LDA as approved intervention: Awareness and use of LDA varies by geography and level of care.

Clinical utility of LDA: LDA reduces most perinatal risk associated with early onset PE. Maternal population risk is associated with the 75%–85% of PE/E that arises at term, so gap still exists even with LDA use. Recent results from ASPIRIN trial suggest that LDA may also reduce risk of preterm birth in nulliparous women (Hoffman et al:2020).

No clear consensus on best screening protocol: Many reputable groups (WHO, NICE, FMF, ACOG, FIGO) recommend somewhat divergent screening protocols in terms of required tests, drug dosage, and when to start and stop administration.

B Health system barriers

Access to ANC: LDA should be started early in pregnancy and most women in LMICs start ANC at 20-24 weeks so the opportunity to intervene has passed. Those that do present do not always get seen since system is overloaded.

Need accurate gestational age for screening protocol: Lack of ultrasound equipment and imaging as part of ANC impedes obtaining accurate gestational age. Pregnant people often do not remember last menstrual period accurately.

LDA seen as invasive drug with risks: Provider concerns around increased susceptibility to bleeding and miscarriage. People don't always like taking drugs while pregnant.

C Refinement of screen

LDA dosage: Concept Founda starting a trial in Ghana, Kenya Africa to compare efficacy of 8 mg LDA for PE/E prevention.

Screening technologies: Feta Foundation free, on-line risk ca provides a clear numerical esti individual risk; women possibly take LDA if given a quantified r

Point of care PLGF lateral flow diagnostic being developed by on their RONIA™ Platform usi blood sample could offer oppo integrate affordable angiogenic test for PE/E into ANC.

NABLERS TO SCALE

A is included in the national policies and is being prescribed routinely in some her-level facilities only

LDA is included in the National Guidelines on Quality Obstetrics and Perinatal Care (2022) and in the Mentor Manual (2022).

Pregnant women are classified as high risk of pre-eclampsia by presence of one or more risk actors (family history, previous history of pre-eclampsia, and chronic disease such as diabetes, kidney disease, and hypertension).

Women identified at high risk should receive aspirin prophylaxis commencing at 11-14 weeks + 6 days of gestation at a dose of ~ 150 mg to be taken every night until either 36 weeks of gestation, when delivery occurs, or when PE is diagnosed.

Estimation of gestation dating mostly is done through EDD and ultrasound. There is recent adoption of technology through the use of point of care ultrasound (POCUS) conducted by nurses and clinicians at lower-level facilities

MOH is incorporating awareness of low dose aspirin in pre- conception care clinics

KEML 2023 guides on LoU for LDA 75 mg at Level 4 and above

“MOH recomme
pregnant wome
are hypertensiv
initiated on L”

Decision maker,

BARRIERS TO SCALE

sk stratification for pre- eclampsia not done early enough, inadequate point of care rasound machines to support gestational age dating

There is low uptake since the medicine is not prescribed at lower facilities

The national policies are not in alignment: The KEML restricts the use of LDA to Level 4 and above, therefore making it not accessible to lower-level facilities.

Risk stratification for pre-eclampsia is a challenge as it happens late. Most mothers have not had their 1st visit at 11 weeks. According to KDHS 2022, 66% of women had 4 or more ANC visits for their most recent live or still birth.

Lack of adequate point of care ultrasound machines in the country to support gestational age (GA) dating.

Inadequate of skills among health care workers for using ultrasound to support GA dating.

Use of ultrasound is usually conducted by radiologists and lacks task shifting policies to support nurses and clinicians undertaking this service.

"There is nowhere t
is working better tha
since it is not bein
out well.
Advocate, Ke

Risk stratification
early, but women d
into ANC that early
stratification (ac
happens much
Advocate, Ke"

rogress toward milestones

ny national policy adoption indicators and training updates have been met, and LDA is widely available at a low cost; however, tra
erage of LDA among pregnant women is needed for monitoring scale up.

bal guidelines & arket availability	National policy adoption	System integration & readiness	Implementation & service delivery	Availability	Coverage
HO Guidelines	**2.1** National SRA/ Regulatory Body Approval	**3.1** Commodity Procurement Specifications	**4.1** Guidelines Disseminated to Subnational-Level	**5.1** Asset Availability Ensured **KHFA 2018: 61% of facilities have aspirin available**	**6.1** Behavior Change of Audience Members
bal Partners	**2.2** Policy Adopted/ Revised for Asset	**3.2** Country LMIS	**4.2** Guidelines Disseminated to Service-Delivery Level		**6.2** Population Coverage Achieved
HO/SRA ated acturers	**2.3** Clinical Guidelines	**3.3** Relevant Indicators Integrated into HMIS	**4.3** HCW Implementation Training	**5.2** Job Aids Available at Facility Level	**6.3** Effective Coverage Achieved
	**2.4** Essential Medicines List	**3.4** Training Curricula Updated	**4.4** Routine Mentorship		**6.4** Equitable Coverage Achieved
	**2.5** Stakeholders Engaged	**3.5** In-service Training Updated	**4.5** Demand Generation Activities		
	**2.6** Costed Implementation Plan				
	**2.7** Budget Line				

ndicators met

ne indicators met

indicators met

rmation vailable*

applicable for asset

*Using the Asset Tracker methodology of conducting literature review of policies and national surveys, and following up with key informant interviews, no information was available for this asset and indicator.

otlight, Photos, & Cost Information

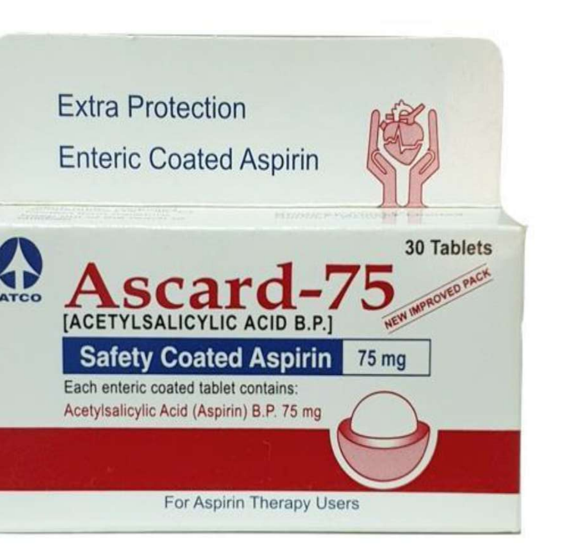

Product: Low Dose Aspirin (Ascard-75)

Pricing:
KES 120 for 30 tablets (KES 4 per tablet / US$0.03 per tablet)

Pricing obtained from a private pharmacy (Source: Janet Shauri)

UNICEF Supply Catalogue Pricing (2023):

Product: Acetylsalicylic acid 75mgtablets, in a blister pack of 56tablets. (Related name -Aspirin)

Pricing:
US$ 0.32 / blister pack
US$ 0.0057 / tablet
US$ 1.12 / full dose

SPOTLIGHT

Concept Foundation will be starting a trial in 3 sites, including Kenya, to compare efficacy of 81 mg vs.150 mg LDA for PE/E prevention

PA

ecommendations for the country to advance scale up

ML revision, curriculum update, dissemination of guidelines, and training of health care workers to support scale u

mediate near-term actions

- Update the KEML to incorporate use of LDA for prevention of pre-eclampsia and lower the level of use (LoU) to level 2 facilities
- Initiate HCW training and mentorship for nurses and clinical officers.
- Disseminate guidelines to sub-county and facility level.

Medium-term actions

- Need for advocacy, awareness generation, a surge efforts to guide the importance.
- Conduct local studies to provide data on effi Disseminate findings to promote awareness usage of LDA
- Include LDA in facility-level budgets and cou plans.
- Increase access of the POCUS machines in lower-level facilities to support gestational da among other things

Nigeria

Methods and data sources

lethods for key informant terviews (KII) and analysis

A structured interview tool was used to guide the discussion, organized along the stages of scale ~~up~~ framework

Virtual and in-person consultations were conducted from June-October 2023 with relevant organizational representatives

Respondents provided verbal consent to participate

Handwritten and electronic notes were expanded, entered in a Smartsheet form, and exported into a framework to support cross-respondent analysis by key themes

Some organizations shared additional programmatic reports, grey literature, and other document resources following KII

List of organizations consulted

- Federal Ministry of Health (FMOH) (Family Health)
- National Primary Health Care Development Agenc (NPHCDA)
- Nigerian Society of Neonatal Medicine (NISONM)/ Neonatal Association
- Clinton Health Access Initiative (CHAI)
- Federal Medical Center, Gombe, Gombe State
- Federal Medical Center, Dutse, Jigawa State
- Management Sciences for Health
- Newborn Essential Solutions and Technology 360
- Nutrition International
- Private Hospital, Kano, Kano State
- Global Financing Facility (GFF)
- Society of Gynaecology and Obstetrics of Nigeria (
- UNICEF
- USAID Integrated Health Program

PA

ata sources summary

- 46 document resources were reviewed including the national Essential Medicines List (nEML), policies, guidelines, and peer reviewed literature, published online and/or hard copies, where available
- HMIS and LMIS data systems and national and global surveys were reviewed for relevant indicators

- Total number of KIIs conducted (n=27), and by type:
  - ❖ Implementers= 16
  - ❖ Advocates= 3
  - ❖ Decisionmakers= 8
- Four interviews were conducted in small groups (FMOH, Nutrition International, CHAI, GFF)

PA

escription of data source documents reviewed

ype of Sources	Total documents (n=46) reviewed across all 8 assets
ebsites	**7** websites, including 1 WHO site; 3 sites for in-country manufacturing; 1 site for in-country TWG; 1 in-country program report; and 2 sites for national guidelines.
lobal Guidelines ocuments	**10** global reports and guidelines
ational Market uthorization reports	**2** documents including NAFDAC Green book and List of devices and NAFDAC Registered Product
ational Policy Documents	**4** National policies including the National Health Strategic Plan 2018-2022, Nigeria Every Newborn Plan, Maternal Health Action Plan, National Policy on MIYC Nutrition in Nigeria
ational Clinical Guidelines Protocols	**10** guidelines and job aids, including CNBC and BNBC guidelines, iCCM CHW guideline and IMCI guidelines and chart booklet, Helping mothers survive guidelines Micronutrient deficiency guideline National ANC guideline and Orientation Package for providers, Shaping Local markets for scale up and ORS in Nigeria, and SOGON Guideline for Management of PPH
ssential Medicine Lists	**3** including Nigeria's EML for Medicines and Drugs, National Medical Equipment List, and WHO's E
HIS / Stockout reports	**2** including Nigeria's DHIS data manual and LMIS stockout report form
ational Surveys	**4** surveys: MIS 2021, NDHS 2018, NNHF 2018, NHFS 2016
raining Curriculums	**4** including the IMCI facilitator and participant manuals, iCCM guidelines, ANC orientation package health providers, and pre-term birth job aids and training booklet

ountry context

uality of maternal and newborn care	DHS 2018	NNHS 2018	MIS 2021
ming of 1st ANC check: less than 4 months	18.3%	Not reported	25.8%
edian months of pregnancy at 1st ANC visit	5.0 months	Not reported	4.7 months
ntenatal visits for pregnancy: 4+ visits	56.8%	Not reported	52.0%
ntenatal visits for pregnancy: 8+ visits	2.0%	Not reported	11.9%
ssistance during delivery from a skilled provider	43.0%	46.0%	Not reported

vailability of Essential Drugs

en though drugs represent an important link in the building blocks of the
lth system and are also central to patients' perception of quality service,
ential drugs which are needed to treat majority of health problems were
vailable in about half of the secondary health facilities and about 7 out
very 10 primary health facilities assessed (NHFS, 2016)

Recommendation
Availability of essential drugs should be mad
priority by government at all levels by providi
resources to strengthen the drug logistics sy
and drug revolving funds (NHFS, 2016)

PA

Results

Calcium Supplementation (CaSupp)

SET DEFINITION: In areas with low dietary calcium intake, pregnant women take 1.5–2.0 g of mental calcium daily, spread over three doses per day for the prevention of pre-eclampsia.

NABLERS TO SCALE

ideline and training updates incorporating calcium supplementation, along with product ailability create an enabling environment

In 2017 the Government of Nigeria tried to adopt the WHO guidelines for provision of calcium for prevention of pre-eclampsia / eclampsia (PE/E), but the process was not operationalized because PE/E was not considered to be a major cause of maternal mortality at that time

The 2018 National Guideline on ANC orientation package recommends that in populations with low dietary calcium intake, daily calcium supplementation (1.5–2.0g oral elemental calcium) be given to pregnant women to reduce risk of PE/E

MOMENTUM 2022 global survey shows that in-service and pre-service training in the public and private sector has been updated to include CaSupp for PE/E but not verified by KIIs

CaSupp is widely available; oral (260 mg) with 1000 mg vitamin C and Sandurst with Vitamin K and D forms can be purchased in private pharmacies

"Now pre-eclamp
prevalent espec
among rural wom
dearth of faciliti
conduct necessar
This is a reason w
government sh
develop a guide
Decision-maker,"

BARRIERS TO SCALE

en used, providers recommend calcium for pregnant women primarily for dietary ficiency, not prevention of PE/E

Providers are not aware that CaSupp is included in the National Guideline on ANC orientation package for health workers

Knowledge of use of CaSupp to prevent pre-eclampsia is very low among providers

nEML(2020) lists calcium salts (carbonate 600 mg tablet and lactate 300mg tablet) are included under "vitamins, minerals, and nutritionals"; does not specify CaSupp for PE/E prevention

In the micronutrient deficiency control guideline (MNDC) (2021), CaSupp for PE/E prevention is not captured

Difficulty in screening and identifying women with low dietary calcium intake

"In Nigeria there i
need for the introd
of CaSupp beca
many people in N
take bones, milk
cheese that are r
calcium."

Implementer, Nig

rogress toward milestones

ny milestones for national policy adoption and system readiness are met as the country focuses on key implementation considera
ance scale; however, there is no availability or coverage data to monitor uptake of calcium supplementation among pregnant wo

bal guidelines & arket availability	National policy adoption	System integration & readiness	Implementation & service delivery	Availability	Coverage
HO Guidelines	**2.1** National SRA/ Regulatory Body Approval	**3.1** Commodity Procurement Specifications	**4.1** Guidelines Disseminated to Subnational-Level	**5.1** Asset Availability Ensured	**6.1** Behavior Change of Audience Members
bal Partners	**2.2** Policy Adopted/ Revised for Asset	**3.2** Country LMIS	**4.2** Guidelines Disseminated to Service-Delivery Level	**5.2** Job Aids Available at Facility Level	**6.2** Population Coverage Achieved
HO/SRA ated acturers	**2.3** Clinical Guidelines	**3.3** Relevant Indicators Integrated into HMIS	**4.3** HCW Implementation Training		**6.3** Effective Coverage Achieved
	**2.4** Essential Medicines List	**3.4** Training Curricula Updated	**4.4** Routine Mentorship		**6.4** Equitable Coverage Achieved
	**2.5** Stakeholders Engaged	**3.5** In-service Training Updated	**4.5** Demand Generation Activities		
	**2.6** Costed Implementation Plan				
	**2.7** Budget Line				

ndicators met

ne indicators met

indicators met

rmation
vailable*

applicable for
asset

*Using the Asset Tracker methodology of conducting literature review of policies and national surveys, and following up with key informant interviews, no information was available for this asset and indicator.

otlight, Photos, & Cost Information

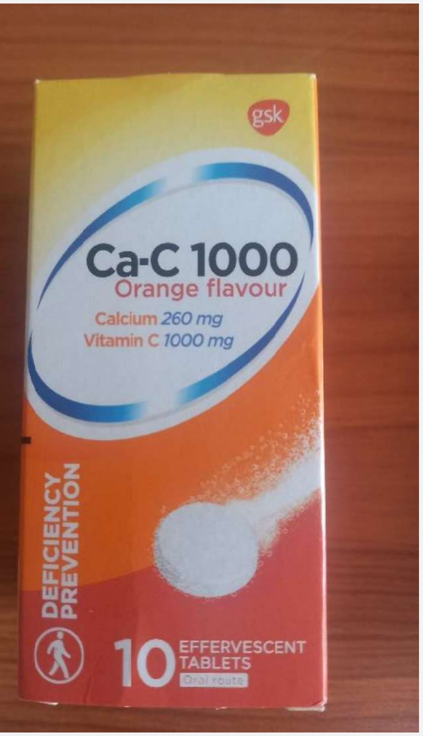

uct: Oral (260mg) Ca-C 1000
ablets)
: N3,000 / US$ 5.35

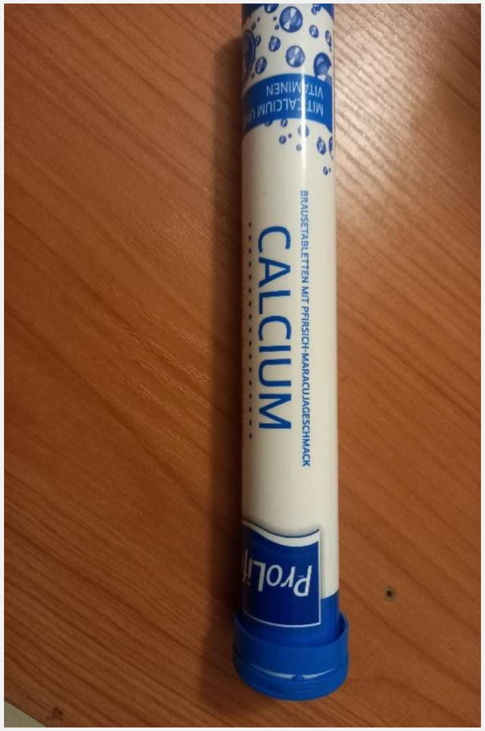

Product: Sandurst CALCIUM (250mg) with vitamin K and D (17 tablets)
Price: N2,250 / US$ 4.01

ative Pricing (UNICEF Supply Catalogue):Calcium Carbonate 1500mg
s,100 tablets / bottle.US$ 2.25 / bottle; US$ 0.0225 / tablet

ce: Obtained from a private pharmacy in Abuja
credits: Gbenga Ishola

SPOTLIGHT

Between 2014-2019, the University of Abuja Teaching Hospital recorded a total of 9,760 deliveries which included 352 women who were managed for PE/E, giving a total prevalence of 3.60% (Akaba et al:2021).

"A local study conducted by staff of the Federal Medical Center Gombe revealed that PE/E and not PPH was responsible for a high number of maternal deaths. This supported a programmatic shift towards provision of Calcium Supplementation in ANC service delivery at Gombe Federal Teaching Hospital." *Decision-maker, Nigeria*

PA

ecommendations for the country to advance scale up

se awareness of calcium use for prevention of PE/E among providers to conform to ANC guidelines

mediate near-term actions

- Raise provider and community awareness about the use of calcium to prevent pre-eclampsia as distinct from use for pregnant women with calcium deficiency
- Include PE/E indication for CaSupp in nEML
- Work with donors and implementing partners to develop training manual and job aids
- Update pre-service and in-service training manual to incorporate the use of CaSupp to prevent pre-eclampsia during pregnancy

Medium-term actions

- Conduct nationwide trainings for health care workers at facility and community level
- Explore a community-directed intervention approach where community health workers (CHWs) provide the asset to increase acce pregnant women, just like misoprostol
- Create procurement specifications and inco into supply chain

PA

ow-dose aspirin
LDA)

SET DEFINITION: Daily low-dose aspirin prophylaxis beginning in the late
t trimester for pregnant women at moderate to high risk of pre-eclampsia.

NABLERS TO SCALE

y stakeholders are engaged on revising guidelines to include LDA for pre-eclampsia evention, LDA has regulatory approval and is widely available for purchase

Aspirin (75mg, 100mg, 300mg) included in nEML list for multiple indications (pain reduction, fever management, and anti-platelet medicine)

In April 2024, FMOH launched a new guideline on safe motherhood (e.g, the Labour Care Guide), which recommends use of LDA for prevention of PE.

Federal Medical Center, Gombe is targeting LDA use in primary health care centers and secondary health facilities, since that is where the majority of women get ANC; screening includes maternal history, blood pressure (BP)

Some implementing partners are incorporating LDA at clinical level; FMOH wants to harmonize disparate guidelines in use; FMOH new safe motherhood guideline includes 75mg LDA for prevention of pre-eclampsia

LDA (75 mg) widely available in private pharmacies

"The Governme
interested in red
pre-eclampsia a
pregnant wome
the country

Decision-maker, I"

BARRIERS TO SCALE

p in policy framework and provider knowledge around screening and use of LDA for e-eclampsia prevention hamper progress toward scale up

Use indication of LDA for PE prevention is not specified in the nEML

The majority of patients that develop pre-eclampsia are in the community

Only 18% women started ANC in the first trimester (NDHS:2018), so screening may not reach all pregnant women

Limited implementation of screening and use of LDA for prevention of PE in in lower-level facilities

Challenge with screening where BP was not taken, or BP was taken and provider did not understand gravity of situation

Limited provider understanding of LDA use beyond high BP management; lack of familiarity with LDA use for pregnant women at risk of PE

Lack of national procurement policy

Generally, there are no data available on LDA at the national level, but data can be obtained from tertiary health facilities where it is being implemented

"Because of the co
on when to comme
use of LDA du
pregnancy and
related considerati
FMOH may opt to
to promote the u
Magnesium sulp
Implementer, N"

rogress toward milestones

ong stakeholder engagement is supporting the push for national policy adoption and will be needed for other key system readines lementation considerations going forward toward scale up.

bal guidelines & arket availability	National policy adoption	System integration & readiness	Implementation & service delivery	Availability	Coverage
HO Guidelines	**2.1** National SRA/ Regulatory Body Approval	**3.1** Commodity Procurement Specifications	**4.1** Guidelines Disseminated to Subnational-Level	**5.1** Asset Availability Ensured	**6.1** Behavior Change of Audience Members
bal Partners	**2.2** Policy Adopted/ Revised for Asset	**3.2** Country LMIS	**4.2** Guidelines Disseminated to Service-Delivery Level	**5.2** Job Aids Available at Facility Level	**6.2** Population Coverage Achieved
HO/SRA ated acturers	**2.3** Clinical Guidelines	**3.3** Relevant Indicators Integrated into HMIS	**4.3** HCW Implementation Training		**6.3** Effective Coverage Achieved
	**2.4** Essential Medicines List	**3.4** Training Curricula Updated	**4.4** Routine Mentorship		**6.4** Equitable Coverage Achieved
	**2.5** Stakeholders Engaged	**3.5** In-service Training Updated	**4.5** Demand Generation Activities		
	**2.6** Costed Implementation Plan				
	**2.7** Budget Line				

l indicators met

ome indicators met

o indicators met

formation navailable*

ot applicable for is asset

*Using the Asset Tracker methodology of conducting literature review of policies and national surveys, and following up with key informant interviews, no information was available for this asset and indicator.

potlight, Photos, & Cost Information

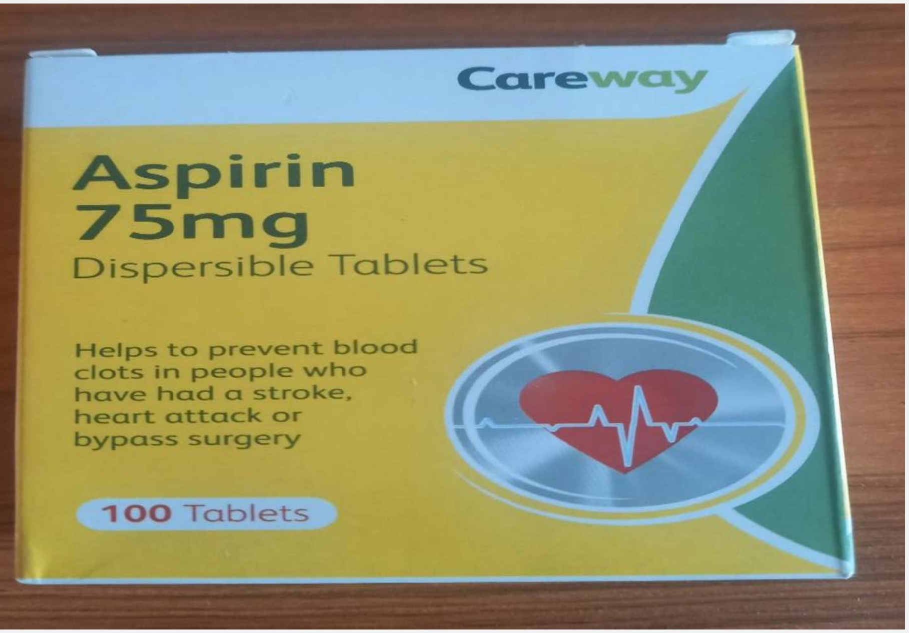

roduct: Aspirin 75mg (100 tablets).
rice: N3,000 / **US$5.37 / pack; US$ 0.054 / tablet**
ource: Obtained at private pharmacy in Abuja
hoto credit: Gbenga Ishola

dicative Pricing (from UNICEF supply catalog):
cetylsalicylic acid 75mgtablets, in a blister pack of 56 tablets.
S$ 0.32 / blister pack; **US$ 0.0057 / tablet**

SPOTLIGHT

"The steps to ensuring scale-up of the asset are to approve its use, including in the nEML, develop training manual, and train service providers from secondary health facilities."

Advocate, Nigeria

PA

ecommendations for the country to advance scale up

ate learning agenda on the effectiveness of LDA in treating pre-eclampsia and development of guidelines and cy supporting use of the asset

mediate near-term actions

- Place LDA on nEML with PE indication
- Update pre-service and in-service training manual to incorporate the use of LDA to prevent pre-eclampsia during pregnancy
- Work with donors and implementing partners to develop training manual and job aids

Medium-term actions

- Conduct nationwide trainings for HCWs at f and community level
- Explore a community-directed intervention approach where community health workers (CHWs) provide the asset to increase acce pregnant women, just like misoprostol
- Create procurement specifications and inco into public sector supply chain

PA

Pakistan

Methods and data sources

lethods for Key Informant nterviews (KIIs) and Analysis

A structured interview guide was used to guide the discussion, organized along the stages of scale up framework

Virtual and in-person consultations were conducted from June–July 2023 with relevant organizational representatives

Respondents provided verbal consent to participate

Handwritten and electronic notes were expanded following the interview, then transcribed electronically and entered in a Smartsheet form, and exported into a framework to support cross-respondent analysis by key themes

Some organizations shared additional programmatic reports, grey literature, and other document resources following the interview

List of organizations consulted

- Ministry of Health (MOH)
- Department of Health
- Lady Health Worker Department (LHW)
- Sindh Healthcare Commission
- Agha Khan University Hospital
- People's Primary Healthcare Initiative
- Maternal, Child Health Center
- Midwifery Association of Pakistan
- Health Services Academy (HSA)
- Greenstar Social Marketing
- Pakistan Institute of Medical Sciences (PIMS)
- Health Planning, Systems Strengthening and Infor Analysis Unit
- Centre of Excellence for Rural Development
- Nutrition International (NI)
- UNFPA
- Japan International Cooperation Agency
- WHO
- UNICEF

escription of data source documents reviewed

of Sources	Total Documents Reviewed across 8 assets
sites	**14** (7 (for global coalitions) + 2 (for DRAP/ in-country manufacturing) + 3 (for in-country TW 1 (for LDA FMF calculator) +1 (online medical store/pharmacy)
al Guidelines Documents	**8** reports
al Market Authorization reports	**4** documents
nal Policy Document(s)	**3** (1 document + 1 (for CIP of ORS-Zinc) + 1 (for maternal nutrition strategy)
nal Clinical Guidelines & Protocols	**12** documents (including, ANC job aids, ANC counselling/complication readiness, IMCI guidelines, clinical guidelines, EmOC)
ational Guidelines Documents	**6** subnational documents (Essential Health Services Packages)
s	**5** EMLs (National, Punjab, Baluchistan, Sindh, & KP)
/ Stockout reports	**3** DHIS reports or manuals (Federal, Sindh, and KP)
nal Surveys	**2** surveys (NNS 2018 & PDHS 2017-18)
ing Curriculums	**4** documents (2 for IMCI facilitator and participant manuals, 1 for ORS-Zinc + 1 LHW traini booklet)

al care; CIP = costed implementation plan; DHIS = District Health Information Software; DRAP = Drug Regulatory Authority of Pakistan; EML = essential medicines list; EmOC = emergency FMF = Fetal Medicine Foundation; IMCI = Integrated Management of Childhood Illness; KP = Khyber Pakhtunkhwa; LDA = low-dose aspirin; LHW = Lady Health Worker; NNS = National y; ORS = oral rehydration salt; PDHS = Pakistan Demographic and Health Survey; TWG = technical working group

ata sources summary

l number of KIIs conducted (n=43), and by type

Stakeholder Type	Number of KII respondents
Advocates	18
Decision-makers	11
Implementers	14
**Total**	**43**

ountry context

uality of maternal and newborn care	PDHS 2013	PDHS 2019
iming of 1st ANC check: less than 4 months	42.4%	**54.6%**
ledian months of pregnancy at 1st ANC visit	3.7	3.4
o ANC visits completed	24.4%	12.2%
ntenatal visits for pregnancy: 4+ visits	36.6%	**51.4%**
ntenatal visits for pregnancy: 8+ visits	Not reported	Not reported
ssistance during delivery from a skilled provider	52.1%	**69.3%**

vailability of Essential Drugs

e Pakistani provincial government is responsible for providing subsidized (or free) medications to government facilities to sure affordable, available treatment for patients. However, when essential medicines are out of stock in government imary, secondary, and tertiary) facilities, patients are forced to purchase drugs from private medical stores and pharmacies.

Results

Calcium Supplementation (CaSupp)

SET DEFINITION: In areas with low dietary calcium intake, pregnant women (PW) are ommended to take 1.5–2.0 g of oral elemental calcium daily spread over three doses per day for prevention of pre-eclampsia. (Note: No set guidance on trimester, providers may wish to mmence CaSupp at first antenatal care [ANC] contact).

OVERVIEW:

CIUM SUPPLEMENTATION upp)

Complicated regimen and conditional global recommendations leaves countries without actionable plans aligning with optimal counseling models for improved adherence; CaSupp is accepte not prescribed.

kground & context

inition: Calcium entation during pregnancy as in areas with low lcium intake, pregnant ke 1.5-2.0 g of elemental aily spread over three r day for the prevention of npsia[1]

ost recent calcium entation during pregnancy was released in 2018

l Guidelines for entation pre-pregnancy n 2020, recommends in research contexts only

obal survey of 31 studying prevention of sive disorders identified 6 untries surveyed included upplementation l guidelines, and 71% nclusion in public pre- nd in-service training.[2]

2 Scale-up status

Global guidelines & market availability (A) → National policy adoption (B) → System integration & readiness → Implementation & service delivery (C) → Availability → Cover

3 Common barriers and enablers to scale

A WHO recommended regimen challenging to meet

Current dosage regimen perceived as high. Current research investigating possibility of impact at lower supplementation levels to reduce required dosage. Latest evidence indicates noninferiority of lower dosage[3]

High dosage leads to logistical challenges Distribution of 3 tablets per day of bulky supplement is logistically challenging without concerted effort and planning

Deviation from WHO in national guidelines Kenya, for example, includes 1.0g/day as the guideline, lower than current recommendation[4]

B Complicated and unclear implementation recommendation

Conditional recommendation focused on low dietary intake of calcium confusing for decision-makers. When dietary information is available, there is limited information on calcium intake, rarely stratified by pregnant women. Global known dietary calcium intake in LMICs is typically lower than recommendations[3]

Definition of low dietary intake is not universal. Different governing bodies prescribe varying levels of optimal dietary calcium intake during pregnancy, typically ranging from 900–1200 mg/day

C Product cost and size barriers to implement

IFA or MMS supplementatio current priority and cheaper, calcium is seen as a cost-bar already challenging ANC imp

Calcium supplements are b creating perceived challeng women with the quantity re Supply chain difficulties for st forecasting supply needs, an

"The easier one makes it [to implement, to take], the more likely it will be used."

Global level, Advocate

NABLERS TO SCALE

nical guidelines exist for PE prevention and management (by SOGP). CaSupp has DRAP proval and is listed on provincial EMLs (primary, secondary care). CaSupp is available OTC or via C visits when women receive nutrition counseling and calcium deficiency testing.

Global Guidelines and Market Availability:

- Past and current initiatives, organizations, and associations are working on prevention of pre-eclampsia (PE) risk using CaSupp and low-dose aspirin (LDA) provision during ANC, including Maternal and Child Health Integrated Program, NI, Action on Pre-eclampsia International, and Pre-eclampsia, Eclampsia Monitoring, Prevention and Treatment (PRE-EMPT)

National Policy Adoption:

- Pakistan has a maternal nutrition strategy 2022–2027 to decrease hypertension and PE via ensuring maternal nutrition by increasing coverage of calcium supplements for pregnant women[1]
- The National pregnancy, childbirth, postpartum and newborn care (PCPNC) essential guide details the clinical nutritional assessment of pregnant women with elevated blood pressure (BP), and the provision of CaSupp (once daily) in the second and third trimester (at the dose of 1.0–1.3 g for daily nutritional intake) (Section C12 & Section F3; page 56 and 104 of the document)

"The MNCH receive
supplements from the
as part of the di
recommendation p
along with other m
and vitamins
Advocate, Punjab P

- Clinical guidelines issued by Society of Gynecologists and Obstetricians of Pakistan (SOGP) on Hypertensive Disorders in Pregnancy (2022) recommend provision of 1.0 g of calcium from the 16th week of pregnancy to prevent risk of pre-term PE
- SOGP and other private organizations have been advocating for prevention and management of pre-eclampsia, including provision of the asset as per the recommendations
- NI is working with the National Committee for Maternal and Neonatal Health (NCMNH) to improve the nutritional status of women and children through community drives and workshops; NI is providing CaSupp to communities via door-to-door visits made by LHWs
- Drug Regulatory Authority of Pakistan (DRAP) approval exists for elemental calcium tablets within calcium carbonate 1250 mg (equal to 500 mg elemental calcium) and calcium lactate (300 mg) tablets
- CaSupp is locally manufactured by various local industries, including GlaxoSmithKline's calcium carbonate 1250 mg (500 mg elemental calcium)
- National and provincial essential medicines lists (EMLs) list calcium lactate tablet at 500 mg; 1 g at the primary and secondary health care level in the vitamin mineral category. Calcium carbonate chewable tablets 1250 mg (500 mg Ca) are only present at the primary level in the Punjab EML. CaSupp is not listed in EMLs for pre-eclampsia/eclampsia (PE/E) prevention.

"CaSupp is an OT
available in the m
*Implementer, Fe
Pakistan*

ystem Integration and Readiness:

- Procurement specifications exist for CaSupp in above-mentioned forms and dosage; supply of the asset to Khyber Pakhtunkhwa (KP) and Punjab is made via the tender-based procurement system, allowing purchase at the lowest possible market price (cost minimization) (KII respondent, Procurement & Supply of Medicines document)[2]
- District Health Information Software (DHIS) includes key indicators reflecting upon the asset such as: nutritional status evaluated during ANC, nutritional counselling during ANC, essential screening tests (BP, urine albumin test)

mplementation Service Delivery:

- In Punjab, Sindh, KP, and Islamabad Capital Territory, pregnant and lactating women are counseled during ANC visits on daily calcium intake[3] (however, CaSupp provision and counseling does not entail PE/E risk prevention) and checked for calcium deficiency*
- Health care workers (HCWs) at health facilities (HFs) are trained on screening for risk of hypertension and PE/E
- LHWs are trained on CaSupp for women with elevated BP and distribute within communities (pg 307 of the LHW training curriculum). All health care providers (HCPs) receive basic training on CaSupp intake as dietary supplements in all regions
- Pregnant women receive CaSupp as a dietary supplement during ANC visits (not specific to PE/E)

"CaSupp is an OT
available in the m
Implementer, Fe
Pakistan

As part of NMNCH's community work in Punjab and KP, medication adherence to once-a-day calcium intake has improved due to door-to-door distribution of calcium supplements

Good understanding among HCPs to administer iron and calcium supplements hours apart than concomitantly to avoid the negative interactions (KII),[4] but it is not written into the SOGP guidelines

vailability of Calcium Supplements:

Calcium supplements are over the counter (OTC) drugs, therefore easily available at primary health care (PHC) centers, maternity centers, and pharmacies

Provision of calcium carbonate as part of the Minimum Service Delivery Package. If CaSupp not provided by facilities, they are prescribed for women to purchase from elsewhere (KII respondent)[5]

Stockouts are minimized in Sindh due to the proper demand calculation mechanisms and forecasting (facilities maintain 15 days of stock)[6]

Basic screening equipment to determine if a pregnant women is at risk of PE, such as BP apparatus are available in HF settings

"CaSupp is an OT
available in the m
Implementer, Fe
Pakistan

overage:

The National Nutrition Survey (NNS, 2018)[7] reports on calcium supplement intake (26.8%) and adherence (17.8% PW took it for 90 or more days, and 62.6% took it daily) by PW across regions in Pakistan by age, education levels, wealth. It also mentions source of advice and provision of CaSupp by skilled providers (received from: OBGYNs 78%, doctors 9%, LHWs 5%, Lady Health Visitors (LHVs) 2%, community health workers (CHWs) 1%, traditional birth attendants 1%)

29% received CaSupp via public sector, 58% received CaSupp via private medical sector, 14% from other sources

"CaSupp is an OT
available in the m
Implementer, Fe
Pakistan

BARRIERS TO SCALE

tional policy framework for PE prevention does not exist. CaSupp 1x daily (not 3x's) is prescribed prevent deficiency. Consistent procurement and supply of CaSupp is adversely affected by the rly tender system. HCPs lack knowledge of importance of CaSupp. Cost is prohibitive and gnant women prefer multivitamins (with Ca) over CaSupp alone.

National Policy Adoption:

The Essential Package of Health Services does not include provision of CaSupp to pregnant women for prevention of PE/E risk

The national emergency obstetric care (EmOC) manual (year not specified) only refers to calcium gluconate for treatment of PE cases. It only hints at provision of CaSupp as a preventative measure for pregnancy induced hypertension, however, it states that its 'beneficial effects have not yet been proven"

Elemental calcium tablets are not mentioned in the EMLs with the specification of prevention of pre-eclampsia; only calcium gluconate injections are listed for treatment of PE/E cases

Clinical guidelines have recently been developed for hypertension disorder during pregnancy, however, they do not entail detailed instructions on provision of CaSupp.
The recommendation is to provide 1 gm of calcium tablet from 16th week of gestation.

ANC guidelines (PCPNC) do not mention prescribing of and counselling on CaSupp to pregnant women for PE/E risk prevention

"The doctor prescribe
policy in Pakistan eve
with pregnancy must b
calcium supplementati
supplement, but for the
of pre-eclampsia the
limited practice, not as
Advocate, Federal

- Lack of clear policy guidelines and recommendations for CaSupp provision to prevent risk of PE/E conclusively results in absence of critical job aids and provision of counseling to PW regarding CaSupp's importance in risk reduction for PE/E
- Costed Implementation Plan (CIP) does not exist for the asset for preventing PE/E risk. However, the maternal nutrition strategy indicates development of a CIP for CaSupp for the indication
- The budget line is dedicated for CaSupp forms mentioned in EMLs, however, these are not specified for PE/E risk prevention
- Trainings on calcium supplements do not include use of supplements for prevention of pre-eclampsia[1]
- Lack of awareness among HCPs regarding the importance of "elemental calcium" supplements[2] with respect to preventing the risk of pre-eclampsia

System Integration and Readiness:

- Commodity procurement specification exists for CaSupp forms (lactate, carbonate), however, CaSupp is not mentioned in EMLs for PE/E risk prevention specification, therefore the procurement specification for the indicated use does not exist

"Mostly elemental
formulation of 400 to
not very easily avai
available] Elemental C
mg costs around 15 ar
to $0.25 USD) total
most cannot afford an
it and some pregnant w
it's not necess
Advocate, Pak

- DHIS does not record data on asset specific indicators such as provision of 1.5-2.0g of elemental calcium carbonate/lactate, daily spread over three doses per day for the prevention of pre-eclampsia, calcium carbonate/lactate available
- Pre- and in-service curricula is not yet updated to recommend and inform on the use of CaSupp for PE/E risk prevention

Implementation Service Delivery:

- There is no data available to assess # of HCWs trained on CaSupp for its use in preventing risk of PE/E
- There are no knowledge assessments conducted with HCWs to gauge their understanding and information on role of CaSupp in preventing risk of PE/E and its accurate dosage for the indication
- While supportive supervision exists, none is determined for provision of asset for the indicated use
- KIIs inform prescribing less dosage of CaSupp compared to the recommendations by WHO due to unavailability of recommended formulations[3]

"Mostly elemental
formulation of 400 to
not very easily avai
available] Elemental C
mg costs around 15 ar
to $0.25 USD) total
most cannot afford an
it and some pregnant v
it's not necess

Advocate, Paki

Availability of Calcium Supplements:

- The available calcium supplements are in form of multivitamins (i.e., the drug/supplement has other active ingredients [e.g., Vitamin D and K[3,4]]) Low availability of 500 mg elemental calcium tablets compared to 200–300 mg doses of elemental calcium.[4] This indicates the need to prescribe dosage broken down into 3 times a day, however, communities resist or fail to adhere to supplements with such dosage requirements[4]
- Chances of stockout in the public sector increase due to the yearly tender-base procurement system and the merger of the health chapter to the central public procurement regulatory authority (PPRA) in KP and Punjab.[5] The process of technical evaluation of bids is time consuming and often is contested by the applicants, causing delays in finalizing the procurement company, thus, leading to supply delays and subsequently stockouts of the asset[5]
- Available dosage in the market that satisfy the 1.5–2.0 g calcium intake requirement for prevention of pre-eclampsia is costly. Cost ineffectiveness of suitable formulations of elemental calcium tablets (for population identified with low dietary calcium intake) hinders community adherence to the supplement

“Mostly elemental
formulation of 400 to
not very easily avai
available] Elemental C
mg costs around 15 ar
to $0.25 USD) total
most cannot afford an
it and some pregnant w
it’s not necess

Advocate, Pak

Coverage:

- Nutritional counseling is part of ANC consultations as mandated by Pakistan PCPNC guidelines. This entails provision of calcium supplementation and counseling for it; however, the counseling is not conducted from the lens of preventing risk of PE/E
- While there is data on PW's calcium intake and adherence, it does not reflect intake and adherence to prevent the risk of PE/E
- Effective and equitable coverage of the asset across rural and urban areas cannot be determined due to lack of regular assessments and data on distribution, uptake and adherence as per WHO recommendation to prevent risk of PE/E[6]

Sustainability:

- The high cost of calcium supplements impacts the daily intake of calcium supplements as per the recommended dosage. Not all regions are covered with the free 3-month provision of calcium supplementation through the Maternal, Newborn, and Child Health (MNCH) Program in Punjab, Sindh, and KP (KII7)
- The continuous availability of calcium supplements is also compromised by diversion of stocks towards disaster-stricken communities, such as the Bill & Melinda Gates Foundation Top-Up Program contribution, made in 2021, which was diverted toward flood affected areas (per IM P 20, KP)[8]

"Mostly elemental
formulation of 400 to
not very easily avai
available] Elemental C
mg costs around 15 ar
to $0.25 USD) total
most cannot afford an
it and some pregnant w
it's not necess
Advocate, Pak

- Sustainable supply of the asset is compromised in Punjab and KP due to the yearly tender-base procurement system per which the selected procurement company supplies the drug to HFs
- KII informed that the stock procurement is an annual procedure and does not account for sudden demand surges. Moreover, absence of buffer stock further exacerbate the situation[9]
- Beneficiaries prefer to receive, or even purchase. CaSupp from private sector as compared to the ones provided in the public sector. Community bias and conception that medicines procured by private sector are of better quality, despite high prices, affects demand and uptake of CaSupp from public health care facilities & practitioners
- Moreover, beneficiaries prefer having supplements that are combination formulas as it takes away the need to purchase, remember, and adhere to multiple medicines and supplements to be taken in a day for extended period of time. This hinders adherence to elemental CaSupp which, as per WHO recommendation, has to be broken down into 3 times a day

"In private practice, al
[practice], the patient
although multinationa
have also been procu
sector by Punjab go
that the combin
[multivitamin] drugs
prescribed in private
more potent or eff
Implementer, Punjab

sset scale up journey

gress toward milestones: Some milestones for national policy adoption and system readiness have been met, but many more are effective implementation. There is limited availability and coverage data to monitor implementation of CaSupp among pregnant w

bal guidelines & arket availability	National policy adoption	System integration & readiness	Implementation & service delivery	Availability	Coverage
HO Guidelines	**2.1** National SRA/ Regulatory Body Approval	**3.1** Commodity Procurement Specifications	**4.1** Guidelines disseminated to subnational level	**5.1** Asset Availability Ensured	**6.1** Behavior Change of Audience Members
bal Partners	**2.2** Policy Adopted/ Revised for Asset	**3.2** Country LMIS	**4.2** Guidelines Disseminated to Service-Delivery Level	**5.2** Job Aids Available at Facility Level	**6.2** Population Coverage Achieved
HO/SRA ated acturers	**2.3** Clinical Guidelines	**3.3** Relevant Indicators Integrated into HMIS	**4.3** HCW Implementation Training		**6.3** Effective Coverage Achieved
	**2.4** Essential Medicines List	**3.4** Training Curricula Updated	**4.4** Routine Mentorship		**6.4** Equitable Coverage Achieved
ndicators met	**2.5** Stakeholders Engaged	**3.5** In-service Training Updated	**4.5** Demand Generation Activities		
ne indicators met					
indicators met	**2.6** Costed Implementation Plan				
rmation available*	**2.7** Budget Line				
applicable for asset					

*Using the Asset Tracker methodology of conducting literature review of policies and national surveys, and following up with key informant interviews, no information was available for this asset and indicator.

ummary of data availability

kistan's scale up of CaSupp for the prevention of pre-eclampsia is characterized with incomplete maternal nutritic icators in the health information system. The data collection is devoid of coverage statistics and availability statis elemental calcium supplements in HFs.

SUMMARY

direct indicators for tracking ium availability or coverage e identified

ect indicators related to ANC cate nearly a tenth of women ived no ANC and roughly half ed ANC during their 1st trimester.

ost one-third of pregnant women their first ANC visit during their 2nd ester, which is later than mmended per guidelines, forcing the importance of nseling on calcium plementation during first visit

INDIRECT INDICATORS

Indicator name	PDHS 2019	PDHS 201
No ANC visits completed	12.2%	24.4%
Antenatal visits for pregnancy: 4+ visits	51.4%	36.6%
Timing of 1st ANC check: less than 4 months	54.6%	42.4%
Median months of pregnancy at 1st ANC visit	3.4m	3.7m

otlight, Photos, & Cost Information

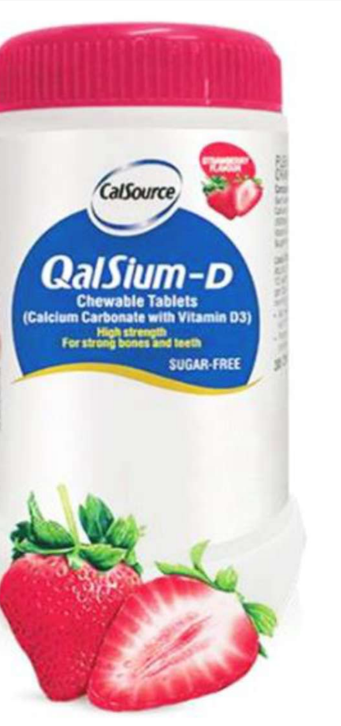

Product: GSK Calcium Carbonate-500mg elemental calcium (GlaxoSmithKline)

Indicative Pricing (UNICEF Supply Catalogue):
Calcium Carbonate 1500mg tablets,100 tablets/bottle.US$ 2.25 / bottle; US$ 0.0225 / tablet

ecommendations for the country to advance scale up

stan needs to revise the national policy on pre-eclampsia prevention to strongly recommend the use of 1.5–2.0 g elemental calcium to match (or "align
) recommendation. This should follow with clinical trials to establish evidence base on the effect of calcium supplement intake in areas with low-calciur
tionally, the training curriculum of LHWs and CMWs should be revised accordingly to educate health practitioners on the importance of elemental calci
ifically for prevention of pre-eclampsia. Lastly, procurement of the drug must be informed with proper demand calculation to minimize the events of sto

mediate near-term actions

Revise national policy to include clear language on pre-eclampsia prevention to include elemental calcium intake of 1.5–2.0 g divided into three doses daily

- Invest in national clinical trials to document the effectiveness of elemental calcium in preventing pre-eclampsia among women living in areas with low calcium intake (prevention vs treatment)

Subsidize the provision of calcium supplements to the rural areas; make multiple micronutrient tablets with CaSupp more available

Engage in community awareness sessions on the importance of elemental calcium for the prevention of pre-eclampsia in areas with reported low-calcium intake

- Enhance community's trust in public health centers to ensure adherence to calcium supplement intake

Training curriculums of LHWs and community midwives (CMWs) needs to be updated to reflect the use of calcium supplements for prevention of pre-eclampsia. Currently, the curriculum only discusses its use as dietary supplement and for bone strengthening

Medium-term actions

In-service trainings for all HCPs on the importance of elem
calcium for pregnant women to prevent the risk of pre-ecla

Integrate data points on the coverage of calcium suppleme
availability through the NNS, general inclusion of maternal
indicators to the health information systems, and adherenc

Set up a proper mechanism for demand calculation and in
buffer stock on a provincial basis

- Invest in a bi-annual requisition process or on a quarterl
on a yearly-base requisition which introduces periods of
- Consider separation of the health chapter from the publ
procurement regulatory authority (PPRA) for timely proc
KP and Punjab, like in Sindh

Effective implementation of the WHO maternal nutrition str
20271 intervention for ANC and postnatal care to speed th
of the asset

PA

Low-dose Aspirin (IDA)

SET DEFINITION: Daily low-dose acetylsalicylic acid (aspirin, 75–81 mg per day) prophylaxis ginning in the late first trimester for PW at moderate to high risk of pre-eclampsia.

OVERVIEW:

-DOSE ASPIRIN (LDA)

Progress toward scale hampered by variable policy framework, low provider and community awareness, late antenatal care attendance and under resourced health systems.

kground & context

inition: Daily low-dose 5-81mg) prophylaxis for women at moderate to of pre-eclampsia by twenty weeks

21 guidance recommends A (75 mg/day) for t moderate and high risk lding on WHO 2011 ndation that focused only n at high risk.

c since majority of me to ANC after 20 DA should be started t; so, most women missing the boat'. a of introducing LDA pp] early is great, so if do that because e not presenting early ow effective is it?"

Implementer, Global level

2 Scale-up status

Global guidelines & market availability (A) → National policy adoption → System integration & readiness (B) (C) → Implementation & service delivery → Availability → Cover

3 Common barriers and enablers to scale

A Barriers to policy implementation

Socialization of LDA as approved intervention: Awareness and use of LDA varies by geography and level of care.

Clinical utility of LDA: LDA reduces most perinatal risk associated with early onset PE. Maternal population risk is associated with the 75%–85% of PE/E that arises at term, so gap still exists even with LDA use. Recent results from ASPIRIN trial suggest that LDA may also reduce risk of preterm birth in nulliparous women (Hoffman et al:2020).

No clear consensus on best screening protocol: Many reputable groups (WHO, NICE, FMF, ACOG, FIGO) recommend somewhat divergent screening protocols in terms of required tests, drug dosage, and when to start and stop administration.

B Health system barriers

Access to ANC: LDA should be started early in pregnancy and most women in LMICs start ANC at 20-24 weeks so the opportunity to intervene has passed. Those that do present do not always get seen since system is overloaded.

Need accurate gestational age for screening protocol: Lack of ultrasound equipment and imaging as part of ANC impedes obtaining accurate gestational age. Pregnant people often do not remember last menstrual period accurately.

LDA seen as invasive drug with risks: Provider concerns around increased susceptibility to bleeding and miscarriage. People don't always like taking drugs while pregnant.

C Refinement of screen

LDA dosage: Concept Founda starting a trial in Ghana, Kenya Africa to compare efficacy of 8 mg LDA for PE/E prevention.

Screening technologies: Feta Foundation free, on-line risk ca provides a clear numerical esti individual risk; women possibly take LDA if given a quantified r

Point of care PLGF lateral flow diagnostic being developed by on their RONIA™ Platform usi blood sample could offer oppo integrate affordable angiogenic test for PE/E into ANC.

NABLERS TO SCALE

tional policy framework needs strengthening to serve as base for LDA use. SOGP nical guidelines exist (without national policy) for providers.

onal Policy Adoption:

e National Comprehensive SRHR Guidelines 2022 recommend the LDA-75mg initiation for women at h-risk of developing pre-eclampsia "before 20 (and if possible as early 12) weeks of gestation for men".

e Pakistan PCPNC Guide (2012) details the screening protocol for pre-eclampsia, and suggests propriate antihypertensive" (e.g., methyldopa, LDA) use when a PW is detected with high BP (90–0mmHg) and 2+ proteinuria.

cent clinical guidelines prepared by SOGP on Hypertensive Disorders in Pregnancy (Baqai et al. 22) recommend 150 mg aspirin for PW with risk score of >1 in 100 on web-based Fetal Medicine undation (FMF) calculator to prevent PE.

e SOGP clinical guidelines refer to the International Federation of Gynecology and Obstetrics (FIGO) delines for first-trimester screening and prevention, which includes early identification of PW at risk of in first trimester (11–14 weeks) using maternal characteristics, mean arterial pressure (MAP) and rine artery pulsatility index (UTA-PI); data is entered into the web based FMF2012 software tool.[1] Risk calculated automatically. For low-resource settings, screening is set for at least 2 parameters (i.e., P and maternal risk factors).

"The BP apparatus
and cheap. Doppler
available, so it is
However, mainter
equipment is an is
machines are outd
digital one show wro
There are defects in
machines but there i
funds to them. There
regular maintenan
machine rather than
only when the dev
working. This is the
Advocate, Sindh

Guidelines Section 5.2: Prevention-to offer tablet aspirin 150 mg at bedtime initiating before 16 weeks and to continue till 36 weeks

ystem Integration and Readiness:

Standardized clinical protocols to screen for the risk of pre-eclampsia during ANC stage exists which examines the blood pressure levels and protein in urine[2]

Providers are aware and follow the screening protocols as part of their routine ANC practice; HFs record PE/E cases

Availability of supportive devices (such as BP apparatus and albumin kit) at all health points to determine pre-eclampsia risk

Private sector: PE risk assessment conducted via FMF, BP, proteinuria tests, and recording maternal history

Public sector: PE risk assessment conducted via FMF in large (district, tertiary) hospitals, BP, proteinuria tests, and recording maternal history

Pre-service and in-service trainings updated to global best practices on use of LDA for PE prevention in high-risk PW (JHPIEGO, 2022)

More than half of women (55%) have their first ANC visit in the first trimester, as recommended (PDHS 2017-18). Most (89%) PW attending ANC have their BP measured (confirmed PE cases are recorded, the number of PW at moderate to high-risk are not recorded)

“A midwife uses RD
check the albumin i
and determine high
on the basis of h
IR+1+2+3; which in
that this woman is g
eclampsia. The kit
color system. If albur
for example, then it i
+2 is yellow, and +3 a
red. If it is red, we in
that albumin is h
Implementer, Sindh I

The existing infrastructure seems favorable in catalyzing prophylactic use of LDA[1] based on:

- Clear SOGP clinical guidelines outline screening protocols for PE/E risk in detail and strong recommendation for LDA prescription (Baqai et al., 2022 link)
- Screening equipment exists in HFs (e.g., BP apparatus, ultrasound facility, UTA-PI dipsticks, and rapid diagnostic test [RDT] albumin kits) with midwives and nurses to assess the level of risk
- Access and knowledge (although limited to providers in private hospitals) of feto-maternal calculators
- Recording of family history of hypertension, maternal/obstetric history from PW, including history of gestational diabetes, and signs of swollen feet
- ANC coverage in the first trimester has improved from 42% (PDHS, 2013) to 55%

"A midwife uses RD
check the albumin i
and determine high
on the basis of h
IR+1+2+3; which in
that this woman is go
eclampsia. The kit
color system. If albur
for example, then it i
+2 is yellow, and +3 a
red. If it is red, we in
that albumin is h
Implementer, Sindh I

Availability of Drug:

- 75–81 mg LDA is DRAP approved, and has locally manufactured variants (e.g., Loprin 75 mg, Ascard 75 mg).
- LDA is a low-cost, OTC drug available in recommended form and dose:
- A pack of 30 tablets of 75 mg Loprin Aspirin (enteric coated) costs PKR 49.96 (US$0.18) (DRAP Registration # 014900)
- A pack of 30 tablets of 75 mg Ascard costs PKR 57.70 (US$0.21) (DRAP Registration #: 016600)
- A pack of 30 tablets of 150 mg Loprin costs PKR 97.13 (US$0.35) (DRAP Registration # 013127)

"A midwife uses RD
check the albumin i
and determine high
on the basis of h
IR+1+2+3; which in
that this woman is g
eclampsia. The kit
color system. If albu
for example, then it i
+2 is yellow, and +3 a
red. If it is red, we i
that albumin is h
Implementer, Sindh

BARRIERS TO SCALE

approved national policy or clinical guidelines, resulting in a lack of formal trainings d job aids for the asset. Disrupted supply chain management due to bottlenecks for ely procurement, and low awareness among the pregnant population.

National Policy Adoption:

- National and provincial EMLs do not specify use of LDA for the indication of pre-eclampsia prevention amongst PW
- There is no costed implementation plan for provision of LDA for the indication to prevent risk of pre-eclampsia amongst PW
- This hinders in-service training programs for HCPs providing ANC, procurement and provision of asset to meet the demand, and availability of screening equipment

System Integration and Readiness:

- HCPs prescribe LDA off-label to PW to prevent risk of PE/E. Absence of accurate forecasting for LDA to for PE prevention affects consistent supply
- Forecasting protocols do not exist, hindering timely and need-based procurement of asset for its multiple use, specifically for LDA (as MNCH priority medicine), thus resulting in periods of stockouts4 and contributes to inequitable distribution of equipment5

"I am learning this
that aspirin in low-
also be used
pregnancy for the
of pre-eclamp

Advocate, Federa

- Health management information system (HMIS) (ANC cards, maternal registers, HF monthly reports) lacks imperative indicators regarding pre-eclampsia and provision of LDA, such as: 'number/ proportion of pregnant women at moderate to high risk of pre-eclampsia' , daily low-dose aspirin provided prophylactically to pregnant women during first-trimester, availability of uterine artery doppler ultrasound, UTA-PI dipsticks.

Implementation Service Delivery:

- In-service trainings for HCPs do not entail components on LDA provision to PW at risk of pre-eclampsia'; KIIs mention 'no need of training for LDA,[6] as it is a simple prescription'; however, lack of trainings and dissemination of standardized procedures/protocols for PE/E screening and LDA provision results in varying screening practices as well as prescriptions for LDA (e.g., inconsistent use of FMF).
- KIIs revealed a lack of awareness among HCPs regarding LDA's use in preventing risk of pre-eclampsia for PW.[7]
- While routine clinical supervisory visits are conducted in HFs, none are carried out for LDA provision to pregnant women at risk of PE.
- Need routine maintenance for existing BP machines and other related equipment.

"We do not hav
included in the
because of the lov
of cases of eclam
have only 1% case
for pre-eclampsia
would you invest
have 90% cases
hemoglobin [anem
invest on th

Implementer,

Pakistan

Availability of Drug:

- Since LDA is not specified for the indicated use of preventing PE/E in the EML, the procurement and supply is restricted for specified use/indications (not distributed at ANC), however the use continues off-label for PE/E risk prevention, thereby resulting in stockouts.
- This is further exacerbated due to lack of demand calculation/forecasting mechanisms for assets in general as well as specifically for LDA (for PE/E).
- LDA 75 mg (public) and 150 mg (private) prescribed for pre-eclampsia due to availability.
- Frequent transfer of personnel in government offices causes absence of critical staff to ensure timely procurement, supply, and distribution of medicines and equipment.[8]
- False supply bottlenecks are introduced to inflate the drug prices by the manufacturers.[9]

"We do not hav
included in the
because of the lov
of cases of eclam
have only 1% case
for pre-eclampsia
would you invest
have 90% cases
hemoglobin [anem
invest on th
Implementer,
Pakistan

Coverage:

- No data available on population coverage, effective coverage, and equitable coverage achieved for LDA
- Demand generation for beneficiaries does not exist, in fact it is affected due to sub-optimal ANC coverage during 1st trimester to timely screen for PE/E and initiate LDA as per global recommendations; HCPs focus more on treatment of PE/E cases given late ANC visits, presence of policy guidelines, inclusion of drugs in EML for management/treatment of PE/E cases, and existence of HMIS indicators to record PE/E cases
- Community skepticism persists regarding use of LDA/aspirin due to its predominant use as a blood thinning tablet for coagulation purposes
- In the Federal region, a KII mentioned that HCPs do not follow the global recommendation to initiate LDA by the late first trimester; most prescribe and administer around the 32nd week when it is too late,[10] this is due to PW attending their 1st ANC in the late 2nd/3rd trimester. This is not advisable and can contribute to postpartum hemorrhage (PPH) risk

"We do not hav
included in the
because of the lov
of cases of eclam
have only 1% case
for pre-eclampsia
would you invest
have 90% cases
hemoglobin [anem
invest on th

Implementer,

Pakistan

sset scale up journey

gress toward milestones: Local stakeholders are helping to further national policy adoption. While LDA is available for other indic
ch more is needed to ensure system readiness and implementation. No availability or coverage indicators exist to monitor progres

obal guidelines & arket availability	National policy adoption	System integration & readiness	Implementation & service delivery	Availability	Coverage
HO Guidelines	**2.1** National SRA/ Regulatory Body Approval	**3.1** Commodity Procurement Specifications	**4.1** Guidelines disseminated to subnational level	**5.1** Asset Availability Ensured	**6.1** Behavior Change of Audience Members
obal Partners	**2.2** Policy Adopted/ Revised for Asset	**3.2** Country LMIS	**4.2** Guidelines Disseminated to Service-Delivery Level	**5.2** Job Aids Available at Facility Level	**6.2** Population Coverage Achieved
HO/SRA ated acturers	**2.3** Clinical Guidelines	**3.3** Relevant Indicators Integrated into HMIS	**4.3** HCW Implementation Training		**6.3** Effective Coverage Achieved
	**2.4** Essential Medicines List	**3.4** Training Curricula Updated	**4.4** Routine Mentorship		**6.4** Equitable Coverage Achieved
ndicators met	**2.5** Stakeholders Engaged	**3.5** In-service Training Updated	**4.5** Demand Generation Activities		
ne indicators met	**2.6** Costed Implementation Plan				
indicators met	**2.7** Budget Line				

rmation available*

applicable for asset

*Using the Asset Tracker methodology of conducting literature review of policies and national surveys, and following up with key informant interviews, no information was available for this asset and indicator.

ummary of data availability

irect indicators of LDA use are available from all three regions. Neither the number of pregnant women at risk of daily intake of LDA prophylaxis for PE is tracked on ANC cards or in DHIS records at the state or federal level.

SUMMARY

indicators of LDA use. Data availability varies by e (primary, secondary, tertiary) and region (Sindh, KP ral).

icators

, and Federal records # of ANC visits, # of cesarean and # of pregnant women on all three levels of care women attending at least 4 or 8 ANC visits is recorded els of care in the ANC cards of all three regions ured in ANC is recorded on the ANC cards for both l KP
ctor: The number of pregnant women with PE/E are at secondary/tertiary level, but # of PW at moderate to of PE at secondary and tertiary level are not recorded ector: The number of women with severe PE/E is not on the national HMIS

r level information is sourced from DHIS, DHS, and alth Registers (i.e., ANC cards). ANC cards include otocols to monitor BP, urine protein levels, and bodily symptoms (e.g., swollen feet), to determine clampsia.

INDIRECT INDICATORS

Quality of maternal and newborn care	PDHS 2019	PDH[illegible]
Timing of 1st ANC check: less than 4 months	54.6%	42[illegible]
Median months of pregnancy at 1st ANC visit	3.4	[illegible]
No (0) ANC visits completed	12.2%	24[illegible]
Antenatal visits for pregnancy: 4+ visits	51.4%	36[illegible]
Blood pressure measured in ANC	89.4%	85[illegible]
Urine sample taken	70.8%	60[illegible]
Blood sample taken	70.4%	55[illegible]
Cesarean sections	22.3%	14[illegible]

otlight, Photos, & Cost Information

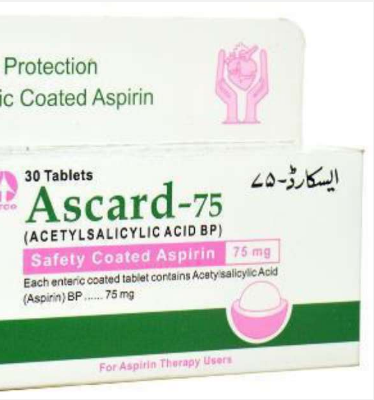

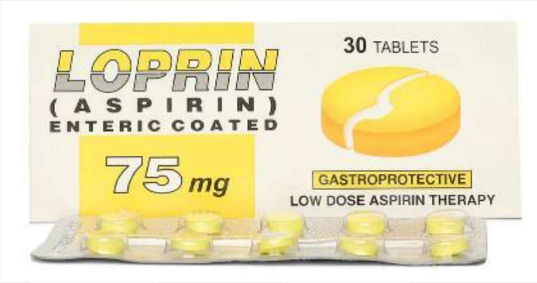

Ascard 75 mg
oated)
ve Pricing:
s
(US$0.21)

Product: Loprin 75 mg (enteric coated)
Illustrative Pricing:
30 tablets PKR 49.96 (US$0.18)

Acetylsalicylic acid 75mgtablets, in a blister pack of 56tablets. (Related
spirin)
e Pricing (from UNICEF Supply Catalog):
/ blister pack
57 / tablet
/ full dose

SPOTLIGHT

Lahore, Pakistan (Anjum et al., 2022)

A random controlled trial enrolled 260 pregnant women between 6- and 32-weeks' gestation to receive daily LDA (80 mg) versus placebo. LDA provided considerable therapeutic effect in preventing pre-eclampsia among high-risk pregnant women ($p<0.001$).

ecommendations for the country to advance scale up

ngthen national policy framework and include LDA in future in-service training curriculums for PHC providers.
ate EML indication of use, and 75 mg LDA bottle labeling to reflect its use for PE prevention. Improve procurement and supply.

mediate near-term actions

- Draft national policy guidelines with strong recommendation for LDA use for PE prevention
- Include LDA (75, 150 mg) in nEML and provincial EMLs with specified use of preventing risk of PE/E
- Revise LDA use/indications on product bottle to include prevention of risk of PE/E (to reduce off-label use)
- Develop a CIP for LDA-use specific to pre-eclampsia prevention
- Develop a demand calculation mechanism for LDA (specifically for PE/E) and related equipment and resources
- At the Federal level, develop rigorous supply chain management systems to address bottlenecks leading to inflated drug prices
- For system readiness, ensure supply of necessary supportive services, such as BP apparatus, doppler, and UTA-PI dipstick; they must be regulated to avoid wastage of resources
- Develop job aids and PE/E screening checklists to ensure fulfillment of screening procedures and accurate prescription of LDA to PW until prophylactic use is approved
- Streamline availability LDA (75–81/150 mg) across private and public health facilities

Medium-term actions

- Train HCPs on: feto-maternal calculator (to ascertain PE risk level), LDA use (to ensure timely identification and administration)
- Include indicators within maternal registers, ANC cards, and facilit reports to record % of PW at risk of PE/E and coverage of LDA
- Catalyze ANC clinics to initiate PW screening protocols to ascertain moderate to high risk of pre-eclampsia
- Increase awareness among pregnant women via participatory community exercises on attending 4+ ANC visits, and the multiple uses of LDA (to reduce skepticism)

PA
